# Acute breathlessness as a cause of hospitalisation in Malawi: a prospective, patient-centred study to evaluate causes and outcomes

**DOI:** 10.1136/thorax-2025-223623

**Published:** 2025-09-10

**Authors:** Stephen A Spencer, Florence Malowa, David McCarty, Elizabeth Joekes, Jacob Phulusa, Beatrice Chinoko, Sylvester Kaimba, Lucy Keyala, Peter Mandala, Mercy Mkandawire, Albert Mukatipa, Mulinda Nyirenda, Hendry R Sawe, Sarah A White, Marc Y R Henrion, Daniel X Augustine, David Oxborough, Eve Worrall, Felix Limbani, Paul Dark, Jamie Rylance, Stephen B Gordon, Ben Morton, On behalf of the Collaborators, Brigitte Denis

**Affiliations:** 1 Department of Clinical Sciences, Liverpool School of Tropical Medicine, Liverpool, UK; 2 Malawi Liverpool Wellcome Research Programme, Blantyre, Malawi; 3 Queen Elizabeth Central Hospital, Blantyre, Malawi; 4 Kamuzu University of Health Sciences, Blantyre, Malawi; 5 Emergency Medicine Department, Muhimbili University of Health and Allied Sciences, Dar es Salaam, United Republic of Tanzania; 6 Royal United Hospitals Bath NHS Foundation Trust, Bath, UK; 7 Liverpool Centre for Cardiovascular Sciences, Liverpool John Moores University, Liverpool, UK; 8 Humanitarian and Conflict Response Institute, University of Manchester, Manchester, UK; 9 Institute of Regeneration and Repair, College of Medicine and Veterinary Medicine, The University of Edinburgh, Edinburgh, UK

**Keywords:** critical care, clinical epidemiology, hypoxemia, pneumonia, respiratory infection, tuberculosis

## Abstract

**Introduction:**

Breathlessness is a common cause of hospital admission globally and is associated with high mortality, particularly in low-income countries. In sub-Saharan Africa, there is a paucity of data on breathlessness, with existing data focused on individual diseases. There is a need for patient-centred approaches to understand interactions between multiple conditions to address population needs and inform health system responses. This multicentre prospective study in Malawi aimed to characterise the aetiologies, outcomes and biomarker accuracy for breathless patients.

**Methods:**

Adults (aged ≥18 years) admitted to medical wards were consecutively recruited within 24 hours of hospital presentation and followed up for 1 year. Participants with breathlessness (defined as a composite of patient-reported shortness of breath; tachypnoea (respiratory rate ≥25/min); hypoxaemia (SpO_2_ <94%) or treatment with oxygen) were systematically screened against internationally accepted diagnostic criteria. We estimated disease prevalence, survival, health-related quality of life and functional status. We also evaluated diagnostic accuracy of natriuretic peptides for heart failure, and procalcitonin (PCT) and C reactive peptide (CRP) for pneumonia.

**Results:**

Of 751 participants, 44% (n=334) had breathlessness, and 316 underwent enhanced diagnostic screening. One-year mortality was higher in breathless patients (51% (157/307)) than those without (26% (100/385)); adjusted HR 1.8 (95% CI 1.4 to 2.3). We identified high prevalence and mortality of heart failure (35% (112/316) prevalence; 69% (75/109) 1-year mortality), anaemia (40% (126/316); 57% (70/122)), pneumonia (41% (131/316); 53% (68/129)) and tuberculosis (29% (91/316); 47% (41/87)). Most participants (63% (199/316)) had multiple conditions. Diagnostic accuracy (area under the curve) for heart failure was 0.89 (brain natriuretic peptide) and 0.88 (N-terminal pro-B-type natriuretic peptide); for pneumonia, CRP was 0.77 and PCT was 0.69.

**Discussion:**

Breathlessness-related hospital admissions in Malawi are common, multifactorial and associated with poor survival. This study demonstrates that co-existing conditions are common, highlighting the limitation of single-disease-focused health system responses. Integrated care pathways with context-sensitive diagnostic and treatment approaches are urgently needed to improve survival.

**WHAT IS ALREADY KNOWN ON THIS TOPIC**

- Previous breathlessness-related research in sub-Saharan Africa has focused on individual diseases.
- No studies have taken a syndromic approach to assess underlying aetiology or captured the complexity or outcomes in patients with breathlessness.

**WHAT THIS STUDY ADDS**

- Our comprehensive, patient-centred, syndromic characterisation of breathlessness demonstrates the considerable burden of breathlessness-related hospital admissions in Malawi.
- Breathless patients frequently suffer multiple coexisting aetiologies and substantial mortality compared with patients without breathlessness.

**HOW THIS STUDY MIGHT AFFECT RESEARCH, PRACTICE OR POLICY**

- This study delivers a step change towards reframing how we understand, manage and design research for breathless patients in low-resource settings.
- Existing health systems and clinical care models traditionally focus on single presenting diseases and do not adequately consider multiple coexisting pathologies.
- Further research is required to develop and evaluate symptom-based integrated acute care pathways, incorporating context-appropriate diagnostic and treatment algorithms to assess their impact on patient outcomes.

## Introduction

 Breathlessness is a common cause of adult hospital admissions globally and is associated with premature mortality.^1 2^ The burden of breathlessness is increasing due to ageing populations, which places significant strain on healthcare systems.^3^ High prevalence of communicable and non-communicable diseases contributes to breathlessness in low-income countries, where diagnostic and treatment logistics are most constrained. The Lancet Commission on Diagnostics,^4^ and 76th World Health Assembly resolutions^5^ highlight the need to strengthen diagnostic capacity and acute care provision in resource-limited settings. In sub-Saharan Africa, aetiology and epidemiology of breathlessness remain poorly characterised,^3^ with a critical gap in knowledge necessary to understand opportunities to develop impactful healthcare interventions and enhance patient outcomes. Our study in Malawi investigates the acute causes of breathlessness-associated hospital presentations, health outcomes and the accuracy of potentially relevant biomarkers to improve diagnosis of common conditions.

Research and clinical care for breathless patients have traditionally focused on specific diseases. However, the recent research priority-setting exercise by the James Lind Alliance—a partnership between patients, carers and clinicians to identify impactful research questions—highlighted the need for a symptom-based research approach for breathless patients to address the rising global burden and increasing prevalence of multiple contributing conditions.^6^ This necessitates symptom-based diagnostic pathways, treatments and intervention development.

Similar evidence-informed approaches have been developed for implementation in primary care, for example, through the Practical Approach to Lung Health and Practical Approach to Care Kit.^7 8^ However, most hospital-based research on this subject in Africa has focused on individual diseases, such as pneumonia.^9^ To our knowledge, a syndromic approach has not been used among hospitalised adults in this setting before. Studies focused on hypoxaemia-related adult admissions in low- and middle-income countries (LMICs) have highlighted a high burden of such patients (10.8% of medical admissions), and high in-hospital mortality (four times higher odds of death compared with those not hypoxaemic).^10^ However, the underlying aetiology, long-term health and functional outcomes have not been addressed. Cost-effective strategies are needed to enhance diagnosis and treatment strategies to improve patient outcomes. In addition, calibration issues with SpO_2_ measurement in people with dark skin are well documented,^11^ but arterial blood gases are frequently not possible in low-resource settings due to expensive capital equipment and running costs. Therefore, a more inclusive approach to identify and manage patients with breathlessness is needed in sub-Saharan Africa.

Our primary aim in this multicentre prospective cohort study was to systematically screen for potential causes of breathlessness among hospitalised adults in Malawi. To our knowledge, detailed examinations of acute breathlessness have not been conducted before in sub-Saharan Africa. Secondary objectives were to measure health outcomes (survival, functional outcomes and health-related quality of life), and evaluate the diagnostic accuracy of biomarkers for common causes of breathlessness. This information is crucial to inform advocacy and priority setting for healthcare delivery in low-resource settings. Our data will also help inform the design of effective health services and development of integrated interventions to improve quality of clinical care and outcomes for acutely ill people in low-resource settings.

## Methods

### Study design

This study was nested within the MultiLink study (*Multimorbidity-associated emergency hospital admissions: a screen and link strategy to improve outcomes for high-risk patients in sub-Saharan Africa*), a prospective observational study investigating multimorbidity among patients admitted to hospital with an acute medical condition in Malawi and Tanzania.^12^ The protocol for the present study has been previously published.^13^ This manuscript adheres to Strengthening the Reporting of Observational Studies in Epidemiology and STARD guidelines (online supplemental tables S1 and S2). Our reflexivity statement (online supplemental file 2) describes how we have promoted equity and capacity building in our international research partnership.^14^

This prospective, multicentre cohort study was conducted in two hospitals in Malawi: Queen Elizabeth Central Hospital, a tertiary referral hospital in Blantyre (1350-bed capacity) and Chiradzulu District Hospital, a district general hospital in Chiradzulu (300-bed capacity). Recruitment was conducted across both sites in parallel and began on 20 September 2022, with 1-year follow-up completed on 12 September 2024.

### Participants

We recruited participants from the MultiLink cohort,^12^ screened at the point of hospital admission and recruited consecutively within 24 hours of emergency presentation. Adults (aged ≥18 years) admitted with an acute medical condition (captured from medical records, using International Classification of Diseases codes) were eligible for enrolment into the MultiLink study.^15^ Participants who experienced breathlessness were eligible for this nested study. Our broad a priori definition of breathlessness^13^ encompassed symptoms and objective physiological parameters, as the presence of at least one of the following: patient-reported shortness of breath, experienced within a 1-week period preceding hospital admission; tachypnoea (respiratory rate ≥25 breaths per minute); hypoxaemia (SpO_2_ <94%) or treatment with supplemental oxygen. Clinical signs were assessed at enrolment into the overarching MultiLink study,^12^ within 24 hours of hospital admission. These physiological threshold levels, based on criteria from UK National Early Warning Score 2,^16^ were chosen to ensure a high-sensitivity inclusion strategy for patients ‘short of breath’. Our inclusive approach aimed to include all patients with breathlessness: both in recognition of the potential issues arising from SpO_2_ calibration^11^; and to include participants whose conditions might not be subjectively perceived as breathlessness but demonstrated by clear objective signs. Full eligibility criteria are outlined in the protocol.^13^ We retained MultiLink^12^ cohort participants who did not meet the breathlessness criteria as a comparator group. These individuals did not undergo enhanced breathlessness-related diagnostics but were screened, recruited and followed up according to our prepublished overarching protocol.^13^

Follow-up assessments were conducted during the hospital stay (days 0, 2, 5, 7 and at discharge), via telephone at day 30, in-person at day 90 and via telephone at 1 year.

### Procedures

All participants were systematically screened for common treatable causes of breathlessness,^13^ including pneumonia, tuberculosis (TB), heart failure, myocardial infarction (MI), anaemia, pulmonary embolism, chronic obstructive pulmonary disease (COPD), asthma, pneumothorax and pleural effusion. A summary of the diagnostic guidelines used for each condition is provided in table 1, with a more comprehensive description of our diagnostic approach in our prepublished protocol^13^ and online supplemental file section 2). Information on diagnostic methodology, quality assurance (QA) and quality control (QC) procedures, and the study schedule is detailed in the protocol.^13^ All tests were conducted and interpreted by experienced and trained personnel (further details are available in the study protocol^13^).

**Table 1:** Conditions and diagnostic guidelines

Condition	Diagnostic guideline (year)
Infection	
Pneumonia	Infectious Diseases Society of America/ATS Consensus Guidelines (2007)^35^
Tuberculosis	Definitions per recent literature (2022, 2023)^36 37^
Cardiac	
Heart failure	Universal Definition of Heart Failure (2021)^22^*
Myocardial infarction	Fourth Universal Definition of Myocardial Infarction (2018)^38^
Obstructive lung diseases	
Chronic obstructive pulmonary disease	ERS/ATS criteria (2022)^39^
Asthma	ATS/ERS criteria (2009)^40^
Haematological/Vascular	
Anaemia	WHO criteria (2011)^41^
Pulmonary embolism	ERS/European Society of Cardiology (2019)^42^ and British Thoracic Society guidelines (2003)^43^
Pulmonary hypertension	British Society of Echocardiography guidelines (2018)^44^
Pleural disease	
Pleural effusion	Radiological and/or sonographic criteria (2020)^45^
Pneumothorax	Radiological and/or sonographic criteria (2010, 2012)^46 47^

Comprehensive details of the diagnostic approach are provided in our a priori protocol^13^ and online supplemental file section 2. Modalities available for use in Malawi and during this study are described in detail in the published protocol.^13^

* Natriuretic peptides, as one of the index tests for the diagnostic accuracy study, were not included as a component of the definition in our study.

ATS, American Thoracic Society; ERS, European Respiratory Society.

Diagnostic tests were conducted at enrolment, with imaging (chest X-ray, ultrasound and echocardiography) performed within 48 hours of admission. Spirometry was conducted at day 90 to allow recovery from acute illness. As part of the overarching MultiLink study, we also screened participants for HIV, hypertension, diabetes and chronic kidney disease.^12^ This full panel of investigations was applied to all enrolled participants.

### Case definition development

We conducted a systematic literature search of diagnostic guidelines to identify internationally accepted case definitions and diagnostic methodologies, applicable to the parameters available in our resource-limited setting. This process, reported within our published protocol, informed the design of our study.^13^ In cases of incomplete diagnostic data, we followed methodological guidance.^17^ Specifically, we (1) identified alternative suitable reference standards and (2) confirmed diagnoses through consensus. For participants without a diagnosis after applying case definitions, their medical notes were reviewed to establish a consensus diagnosis. Full details, including the proportion of participants diagnosed for each category, are in online supplemental tables S3–S16.

### Outcomes

The primary objective was to delineate the causes of breathlessness. Secondary objectives included assessment of patient outcomes captured throughout the follow-up period. These included: readmission rate, hospital length of stay and mortality. We also assessed patient-reported outcomes throughout follow-up, including health-related quality of life (HRQoL; assessed using the Malawi Chichewa language version of the EQ5D-5L (EuroQol Research Foundation; Registration ID: 45352) and functional status (New York Heart Association classification), modified Medical Research Council (mMRC) dyspnoea scale). Indexed HRQoL utility scores were calculated using the Ugandan value set,^18^ as no Malawi-specific value set is currently available.

### Clinical severity scoring

Illness severity on admission was quantified using the Universal Vital Assessment (UVA) score, a validated early warning tool for acute illness in sub-Saharan Africa.^19^ Frailty was assessed using the Clinical Frailty Scale.^20^ Disability was measured using the Washington Group-Short Set on Functioning, and defined as ‘a lot of difficulty’ in at least one domain.^21^

### Diagnostic accuracy study

We evaluated the diagnostic accuracy of biomarkers, including brain natriuretic peptide (BNP) and N-terminal pro-B-type natriuretic peptide (NT-proBNP), for heart failure diagnosis; C reactive peptide (CRP) and procalcitonin (PCT) for pneumonia. Samples were collected at the first study visit. Abbott iSTAT point-of-care BNP (Illinois, USA) was conducted by research staff following comprehensive training. NT-proBNP, CRP and PCT assays were conducted in the Malawi-Liverpool-Wellcome Laboratory, Malawi and the John Hopkins Research Project Laboratory, Malawi, following their quality-assured and quality-controlled laboratory procedures. Further details of assays and laboratory procedures are available in the study protocol.^13^ We assessed the general discriminability of the biomarkers and diagnostic performance against recommended cut-points: BNP at 100 pg/mL and NT-proBNP at 300 pg/mL for acute heart failure^22^; PCT at 0.25 ng/mLand CRP at 25 mg/L for pneumonia.^23^ Investigators who interpreted reference tests required for the diagnosis of heart failure (ie, echocardiography) and pneumonia (ie, CXR and lung ultrasound) diagnoses were blinded to index test results (BNP/NT-proBNP and CRP/PCT, respectively), and those handling index tests were blinded to reference test results (full diagnostic criteria are available in online supplemental tables S3 and S7).

### Statistical analysis

For the primary outcome, to estimate prevalence of at least 20% with 5% precision (margin of error) and α=0.05, the target sample size was 246 participants, inflated to 308 participants to account for 20% inability to confirm diagnosis and/or loss to follow-up.^13^ Categorical variables were summarised as counts and percentages, and continuous variables as medians and IQR or means and SD, depending on data distribution. Follow-up time was from admission to death or censoring. Kaplan-Meier plots summarised mortality, with HRs calculated by Cox regression, or flexible parametric survival models used when proportional hazards assumptions were not met. Model details and flexible parametric model fit are presented in online supplemental file section 6 and figure S5. We also used flexible parametric models to predict survival with adjustment for coexisting conditions to estimate their overall impact. Survival models adjusted for: age, sex, UVA and coexisting conditions with ≥10 deaths/variable (heart failure, pneumonia, anaemia and TB), excluding pleural effusion and pulmonary hypertension as they were most commonly secondary to heart or lung disease. We also assessed the impact of functional and patient-reported outcome measures on mortality. For these exploratory analyses, we used the Benjamini-Hochberg method to adjust for multiple comparisons across univariable survival models. Diagnostic accuracy was assessed via area under the curve (AUC), sensitivity, specificity, predictive values and decision curve analyses using internationally accepted cut-points (exploratory cut-points provided in the online supplemental tables S38 and S39).^22 23^ Since <5% of records had incomplete data for primary or secondary analyses, complete case analysis was used. Analyses were conducted in Stata MP V.18.0 (StataCorp, USA); figures were generated in R V.4.4.1.

## Results

**Figure 1:**
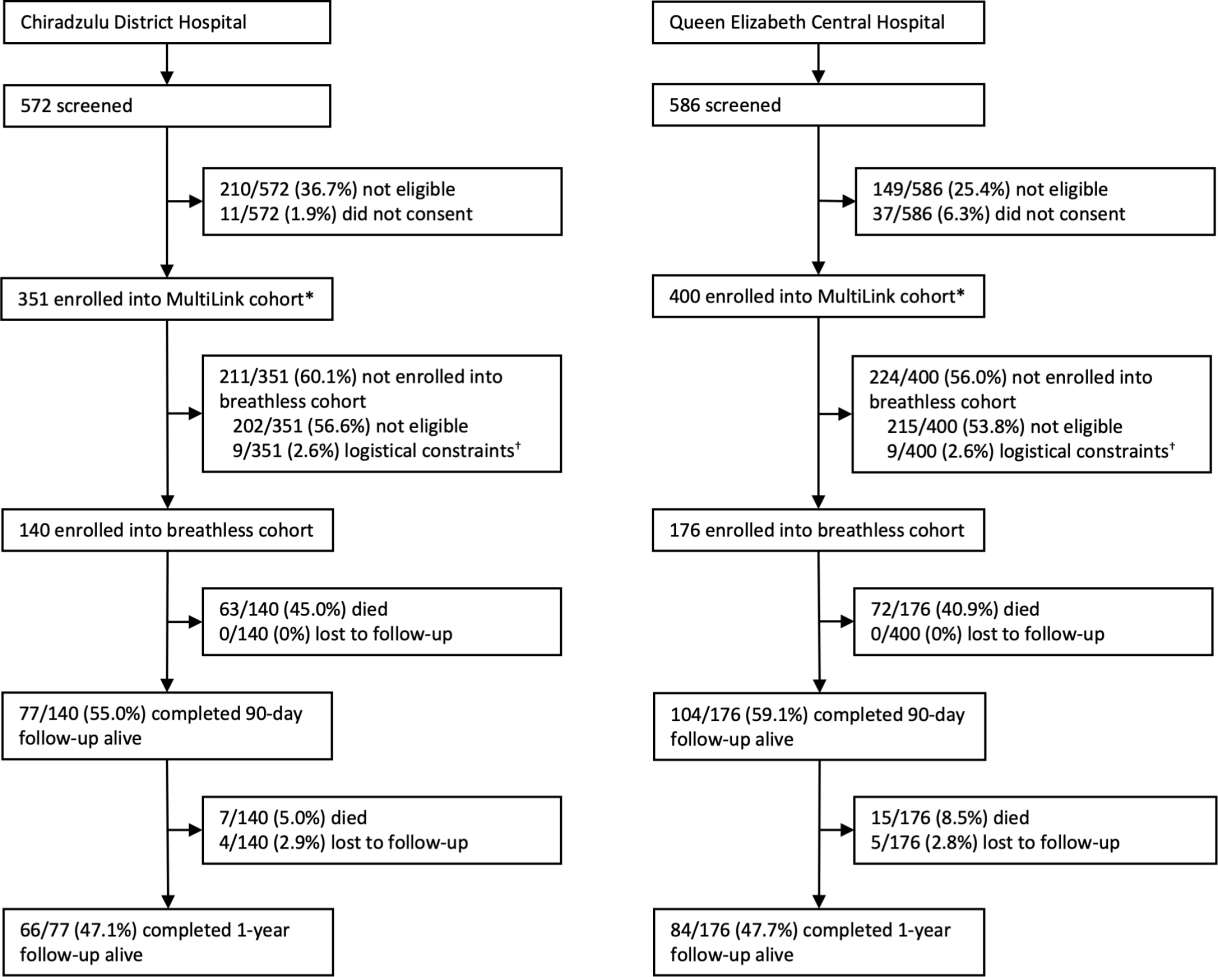
Study profile. Participants were followed up throughout the study. *Our study was a nested component of the MultiLink study, a prospective observational study aimed at identifying multimorbidity among hospitalised medical patients in Malawi and Tanzania (protocol published previously^15^). The non-breathless participants were also followed up to 1 year postadmission as the comparator population, with 1-year outcome status available for 385/417 (92.3% participants). ^†^Logistical constraints: enrolment in the breathless cohort study was temporarily paused due to limitations in staffing or the unavailability of necessary equipment.

We consecutively recruited 751 adults admitted to hospital with acute medical conditions in Malawi within the MultiLink study. Among these, 44% (n=334) met criteria for breathlessness, and 316 were enrolled into the breathless cohort (figure 1); 36% (113/316) female; mean age 51.7 years (SD: 18.1; table 2). One-year outcomes were available for 97% (307/316) participants and 51% (157/307) died during follow-up. The overlap between the constituent components of our breathlessness definition is presented in online supplemental figure S2. Through a sensitivity analysis, we have demonstrated no significant difference in 1-year mortality between constituent components of our breathlessness definition (online supplemental table S18).

**Table 2:** Participant characteristics^16^

	Chiradzulu	QECH	Total
MultiLink cohort*, n	351	400	751
Mean age, years (SD)	48.8 (18.2)	45.2 (17.0)	46.8 (17.7)
Female	170/351 (48%)	142/400 (35.5%)	312/751 (42%)
Male	181/351 (52%)	258/400 (64.5%)	439/751 (58%)
Breathless subcohort, n	140	176	316
Mean age, years (SD)	53.9 (18.4)	50.0 (17.7)	51.7 (18.1)
Female	54/140 (39%)	59/176 (34%)	113/316 (36%)
Male	86/140 (61%)	117/176 (66%)	203/316 (64%)
Employment status			
Unemployed	126/140 (90%)	124/176 (70%)	250/316 (79%)
Student	1/140 (1%)	1/176 (1%)	2/316 (1%)
Housework	0/140 (0%)	2/176 (1%)	2/316 (1%)
Informal paid work	1/140 (1%)	15/176 (9%)	16/316 (5%)
Formal employment	10/140 (7%)	22/176 (12%)	32/316 (10%)
Retired	2/140 (1%)	12/176 (7%)	14/316 (4%)
Education			
Nursery	0/140 (0%)	11/176 (6%)	11/316 (3%)
Primary	98/140 (70%)	86/176 (49%)	184/316 (58%)
Secondary or higher education	35/140 (25%)	72/176 (41%)	107/316 (34%)
Unknown	7/140 (5%)	7/176 (4%)	14/316 (4%)
Mid-upper arm circumference, mean cm (SD)	26.1 (3.3)	24.7 (3.1)	25.3 (3.2)
Current tobacco smoker	10/140 (7%)	22/176 (12%)	32/316 (10%)
Current alcohol use	21/140 (15%)	39/176 (22%)	60/316 (19%)
Symptoms and physiological parameters			
Acute dyspnoea	126/140 (90%)	151/176 (86%)	277/316 (88%)
Tachypnoeic (RR ≥24)	75/140 (54%)	140/176 (80%)	215/316 (68%)
Hypoxaemic (SpO_2_ <94%)	32/140 (23%)	57/176 (32%)	89/316 (28%)
Supplemental oxygen therapy	40/140 (29%)	111/176 (63%)	151/316 (48%)
Number of days since onset of acute illness, median (IQR)	14 (4–31)	7 (3–21)	7 (4–21)
CFS			
Median (IQR)	4 (3–6)	6 (4–6)	5 (4–6)
Not frail (<5)	76/140 (54%)	48/176 (27%)	124/316 (39%)
Frail (5–6)	40/140 (29%)	97/176 (55%)	137/316 (43%)
Severely frail (CFS ≥7)	24/140 (17%)	31/176 (18%)	55/316 (17%)
UVA score			
Median (IQR)	2 (0–3)	2 (1–4)	2 (1–4)
Low risk (0–1)	51/140 (36%)	48/176 (27%)	99/316 (31%)
Medium risk (2–4)	75/140 (54%)	95/176 (54%)	170/316 (54%)
High risk (>4)	14/140 (10%)	33/176 (19%)	47/316 (15%)
Comorbidities			
Hypertension	68/140 (49%)	85/176 (48%)	153/316 (48%)
Diabetes	22/140 (16%)	37/176 (21%)	59/316 (19%)
Chronic kidney disease	5/140 (4%)	17/176 (10%)	22/317 (7%)
HIV infection	45/140 (32%)	71/176 (40%)	116/316 (37%)
Unknown status	0/140 (0%)	4/176 (2%)	4/316 (1%)
HIV control at baseline			
Undetectable viral load (<50 copies/mL)	24/140 (17%)	34/176 (19%)	58/316 (18%)
Controlled (50–199 copies/mL)	2/140 (1%)	2/176 (1%)	4/316 (1%)
Uncontrolled (200–999 copies/mL)	3/140 (2%)	3/176 (2%)	6/316 (2%)
Very poor control (1000–9999 copies/mL)	1/140 (1%)	2/176 (1%)	3/316 (1%)
High infectivity (≥10 000 copies/mL)	8/140 (6%)	8/176 (5%)	16/316 (5%)
New diagnosis	3/140 (2%)	4/176 (2%)	7/316 (2%)
Unknown HIV control	4/140 (3%)	22/176 (12%)	26/316 (8%)
Causes of breathlessness			
Pneumonia	44/140 (31%)	87/176 (49%)	131/316 (41%)
TB	31/140 (22%)	60/176 (34%)	91/316 (29%)
Newly diagnosed†	28/140 (20%)	53/176 (30%)	81/316 (26%)
Known diagnosis	3/140 (2%)	7/176 (4%)	10/316 (3%)
Not diagnosed	109/140 (78%)	116/176 (66%)	225/316 (71%)
History of TB	18/140 (13%)	32/176 (18%)	50/316 (16%)
Post-TB lung disease	6/140 (4%)	13/176 (7%)	19/316 (6%)
Insufficient imaging available	17/140 (12%)	17/176 (10%)	34/316 (11%)
Heart failure	51/140 (36%)	61/176 (35%)	112/316 (35%)
Acute myocardial infarction	2/140 (1%)	6/176 (3%)	8/316 (3%)
Chronic obstructive pulmonary disease	11/140 (8%)	10/176 (6%)	21/316 (7%)
Asthma	14/140 (10%)	4/176 (2%)	18/316 (6%)
Anaemia	57/140 (41%)	69/176 (39%)	126/316 (40%)
Pulmonary embolism	0/140 (0%)	1/176 (1%)	1/316 (0%)
Pneumothorax	1/140 (1%)	2/176 (1%)	3/316 (1%)
Pulmonary hypertension	22/140 (16%)	19/176 (11%)	41/316 (13%)
Precapillary	8/140 (6%)	6/176 (3%)	14/316 (4%)
Postcapillary	14/140 (10%)	13/176 (7%)	27/316 (9%)
Pleural effusion	56/140 (40%)	62/176 (35%)	118/316 (37%)
Suspected malignancy (primary or secondary)	4/140 (3%)	5/176 (3%)	9/316 (3%)
Insufficient imaging available	17/140 (12%)	17/176 (10%)	34/316 (11%)
Suspected interstitial lung disease	2/140 (1%)	7/176 (4%)	9/316 (3%)
Insufficient imaging available	17/140 (12%)	17/176 (10%)	34/316 (11%)

Baseline demographic characteristics, conditions and patient outcomes, by study site.

Figures represent only those with high probability of pulmonary hypertension, based on the British Society of Echocardiography criteria (breakdown shown in the online supplemental table S21).

* Our study was a nested component of the MultiLink study, a prospective observational study aimed at identifying multimorbidity among hospitalised medical patients in Malawi and Tanzania (protocol previously published^15^).

† TB diagnosis based on a composite of clinical, radiological and microbiological data.^13^

CFS, Clinical Frailty Scale; QECH, Queen Elizabeth Central Hospital; TB, tuberculosis; UVA, Universal Vital Assessment.

**Figure 2:**
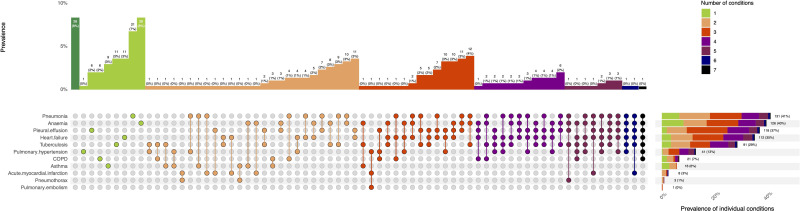
Causes of breathlessness. Aetiologies of breathlessness and their prevalence among patients admitted to hospital in Malawi. The x-axis shows the conditions included. Numbers represent count, n and percentage (%). Where single circles are shown, the corresponding vertical bar chart shows the prevalence of the single condition. Where there are two or more circles, the corresponding vertical bar chart shows the prevalence of coexisting conditions. For example, above the green filled circle for pneumonia alone, the vertical bar shows the prevalence of participants with pneumonia alone (7% (21/316), ie, participants do not have additional conditions); above the orange filled circles for both pneumonia and anaemia, the vertical bar chart shows the prevalence of participants with co-existent pneumonia and anaemia (3% (10/316)). The horizontal bar chart shows the prevalence of participants with each individual condition, stratified by colour to depict the number of additional co-existent conditions. COPD, chronic obstructive pulmonary disease.

Prevalence data for diagnoses identified in the cohort are summarised in figure 2, which shows interactions between coexisting pathologies. This figure describes single, dual and multiple diagnoses. For example, 63% of patients (199/316) had two or more diagnoses associated with their breathlessness. We identified pneumonia in 41% of participants (95% CI 36% to 47%; 131/316); anaemia in 40% (34% to 46%; 126/316); heart failure in 35% (30% to 41%; 112/316) and TB in 29% (24% to 34%; 91/316). Most breathless participants (82%; 259/316) had one or more of these four conditions. We identified 37% (32% to 43%; 118/316) with pleural effusions. Among these, 92% (109/118) were associated with heart failure, pneumonia or TB-related disease processes (online supplemental figure S4). We have disaggregated diagnostic pathways per individual condition in online supplemental tables S3–S16. Pairwise prevalence data are presented in online supplemental figure S1. Prevalence data disaggregated by HIV status are presented in online supplemental tables S30. Eight per cent of participants (26/316) did not meet the criteria for our preselected conditions (clinical consensus diagnoses for these 26 participants are provided in online supplemental table S17). HIV infection was identified in 37% (116/316) of participants; associated with pneumonia (47%; 62/131); TB (62%; 56/91); heart failure (25%; 28/112) and COPD (24%; 5/21), respectively.

We confirmed microbiological aetiology in 27% of pneumonia cases (36/131), including *Mycobacterium tuberculosis* in 16% (21/131); *Streptococcus pneumoniae* in 2% (3/131); *Staphylococcus aureus* in 1% (1/131) and viral pathogens in 8% (11/131), including SARS-CoV-2 in 3% (4/131) (table 3; online supplemental table S19). The overlap between TB and pneumonia diagnoses, including clinical and radiological TB diagnoses, is presented in further detail in the online supplemental figure S3. Cardiac abnormalities were identified in 60% (183/304) of participants: these included left ventricular systolic dysfunction in 26% (80/304); diastolic dysfunction in 37% (113/304) and right heart failure in 21% (72/304). The most common structural cardiac abnormalities were clinically significant valvular disease (33% (99/304); online supplemental table S20 for further details), of which rheumatic valvular disease was identified in 5% (15/304); dilated cardiomyopathy in 16% (50/304); hypertensive heart disease in 16% (50/304) and pericardial effusions in 15% (46/304). All eight cases of acute MI in our cohort were identified as type 2 MI (secondary to mismatched myocardial oxygen supply and demand), with no instances of type 1 MI (secondary to atherosclerotic disease) identified.

**Table 3:** Disease subclassification

	Chiradzulu	QECH	Total
Pneumonia	44/140 (31%)	87/176 (49%)	131/316 (41%)
Pneumonia aetiology by microbiology			
Pneumonia with bacterial pathogen*	0/140 (0%)	4/176 (2%)	4/316 (1%)
Pneumonia with viral pathogen	6/140 (4%)	5/176 (3%)	11/316 (3%)
Pneumonia with microbiologically confirmed TB†	7/140 (5%)	14/176 (8%)	21/316 (7%)
Pneumonia, with no positive microbiology	31/140 (22%)	64/176 (36%)	95/316 (30%)
TB	31/140 (22%)	60/176 (34%)	91/316 (29%)
Pulmonary TB‡	24/140 (17%)	50/176 (28%)	74/316 (23%)
Disseminated TB‡	15/140 (11%)	11/176 (6%)	26/316 (8%)
Cardiac abnormalities§			
Cardiac structural abnormality	84/134 (63%)	99/170 (58%)	183/304 (60%)
HFrEF	33/134 (25%)	32/170 (19%)	65/304 (21%)
HFmrEF	8/134 (6%)	7/170 (4%)	15/304 (5%)
HFpEF	10/134 (7%)	20/170 (12%)	30/304 (10%)
LV diastolic dysfunction	57/134 (43%)	56/170 (33%)	113/304 (37%)
LVDF with normal filling pressures	28/134 (21%)	27/170 (16%)	55/304 (18%)
LVDF with elevated filling pressures	29/134 (22%)	29/170 (17%)	58/304 (19%)
Valvular disease	47/134 (35%)	52/170 (31%)	99/304 (33%)
Rheumatic valvular disease	10/134 (7%)	5/170 (3%)	15/304 (5%)
Dilated cardiomyopathy	22/134 (16%)	28/170 (16%)	50/304 (16%)
Hypertensive heart disease	25/134 (19%)	25/170 (15%)	50/304 (16%)
Restrictive cardiomyopathy	2/134 (1%)	0/170 (0%)	2/304 (1%)
Ischaemic cardiomyopathy	2/134 (1%)	1/170 (1%)	3/304 (1%)
Hypertrophic cardiomyopathy	2/134 (1%)	0/170 (0%)	2/304 (1%)
Constrictive pericarditis	1/134 (1%)	1/170 (1%)	2/304 (1%)
Pericardial effusion	25/134 (19%)	21/170 (12%)	46/304 (15%)
Small	21/134 (16%)	18/170 (11%)	39/304 (13%)
Moderate	2/134 (1%)	1/170 (1%)	3/304 (1%)
Large	2/134 (1%)	2/170 (1%)	4/304 (1%)
RHF	30/134 (22%)	32/170 (19%)	72/304 (21%)
RHF secondary to LHF	23/134 (17%)	22/170 (13%)	45/304 (15%)
Isolated RHF	7/134 (5%)	10/170 (6%)	17/304 (6%)
Cardiac arrhythmia¶	10/135 (7%)	4/172 (2%)	14/307 (5%)
Atrial fibrillation	8/135 (6%)	4/172 (2%)	12/307 (4%)
Atrial flutter	2/135 (1%)	0/172 (0%)	2/307 (1%)
MI	8/140 (6%)	10/176 (6%)	18/316 (6%)
Acute MI	2/140 (1%)	6/176 (3%)	8/316 (3%)
Type 1	0/140 (0%)	0/176 (0%)	0/316 (0%)
Type 2	2/140 (1%)	6/176 (3%)	8/316 (3%)
Previous MI	6/140 (4%)	6/176 (3%)	12/316 (4%)
COPD¶	11/140 (8%)	10/176 (6%)	21/316 (7%)
Mild impairment	4/135 (3%)	2/172 (1%)	6/307 (2%)
Moderate impairment	3/135 (2%)	1/172 (1%)	4/307 (1%)
Severe impairment	2/135 (1%)	3/172 (2%)	5/307 (2%)
Anaemia¶	57/140 (41%)	69/176 (39%)	126/316 (40%)
Mild	19/132 (14%)	25/171 (15%)	44/303 (15%)
Moderate	20/132 (15%)	29/171 (17%)	49/303 (16%)
Severe	9/132 (14%)	5/171 (8%)	14/303 (11%)
Transfusion threshold (Hb <70 g/L)	9/132 (7%)	9/171 (5%)	18/303 (6%)
Pleural effusion¶	56/140 (40%)	62/176 (35%)	118/316 (37%)
Simple effusion	51/136 (38%)	56/174 (32%)	107/310 (35%)
Complex effusion	5/136 (4%)	6/174 (3%)	11/310 (4%)
Pleural effusion aetiology**			
Heart failure-associated effusion	37/140 (26%)	38/176 (22%)	75/316 (24%)
Parapneumonic effusion	19/140 (14%)	39/176 (22%)	58/316 (18%)
TB-associated pleural effusion	12/140 (9%)	25/176 (14%)	37/316 (12%)

Criteria for disease subclassifications are available in our prepublished protocol.^13^ Data within this table are not mutually exclusive.

* Determined by blood culture. Sputum culture and urinary antigen testing were not conducted.

† Microbiologically TB diagnosis was based on Xpert TB or urine-LAM. Clinical and/or radiological TB-associated pneumonia diagnoses in the absence of positive microbiology were also identified in 29/316 participants. The overlap between TB and pneumonia diagnoses are presented in further detail in the online supplemental figure S3.

‡ Pulmonary TB was based on a composite of a microbiological (Xpert TB positivity), clinical and/or radiological diagnosis (with disaggregated data provided in the online supplemental table S19). Disseminated TB was determined by urine-LAM positivity.

§ Echocardiography conducted on 304 participants.

¶ Diagnostic definition met according to a priori criteria. Subclassification not possible due to limited data as indicated in the table (further details available in the online supplemental tables S19–S26).

** The overlap between pleural effusion aetiologies is presented in further detail in the online supplemental figure S4.

COPD, chronic obstructive pulmonary disease; Hb, haemoglobin; HFmrEF, heart failure with mildly reduced ejection fraction; HFpEF, heart failure with preserved ejection fraction; HFrEF, heart failure with reduced ejection fraction; LAM, lipoarabinomannan; LHF, left heart failure; LV, left ventricle; MI, myocardial infarction; RHF, right heart failure; TB, tuberculosis .

**Figure 3:**
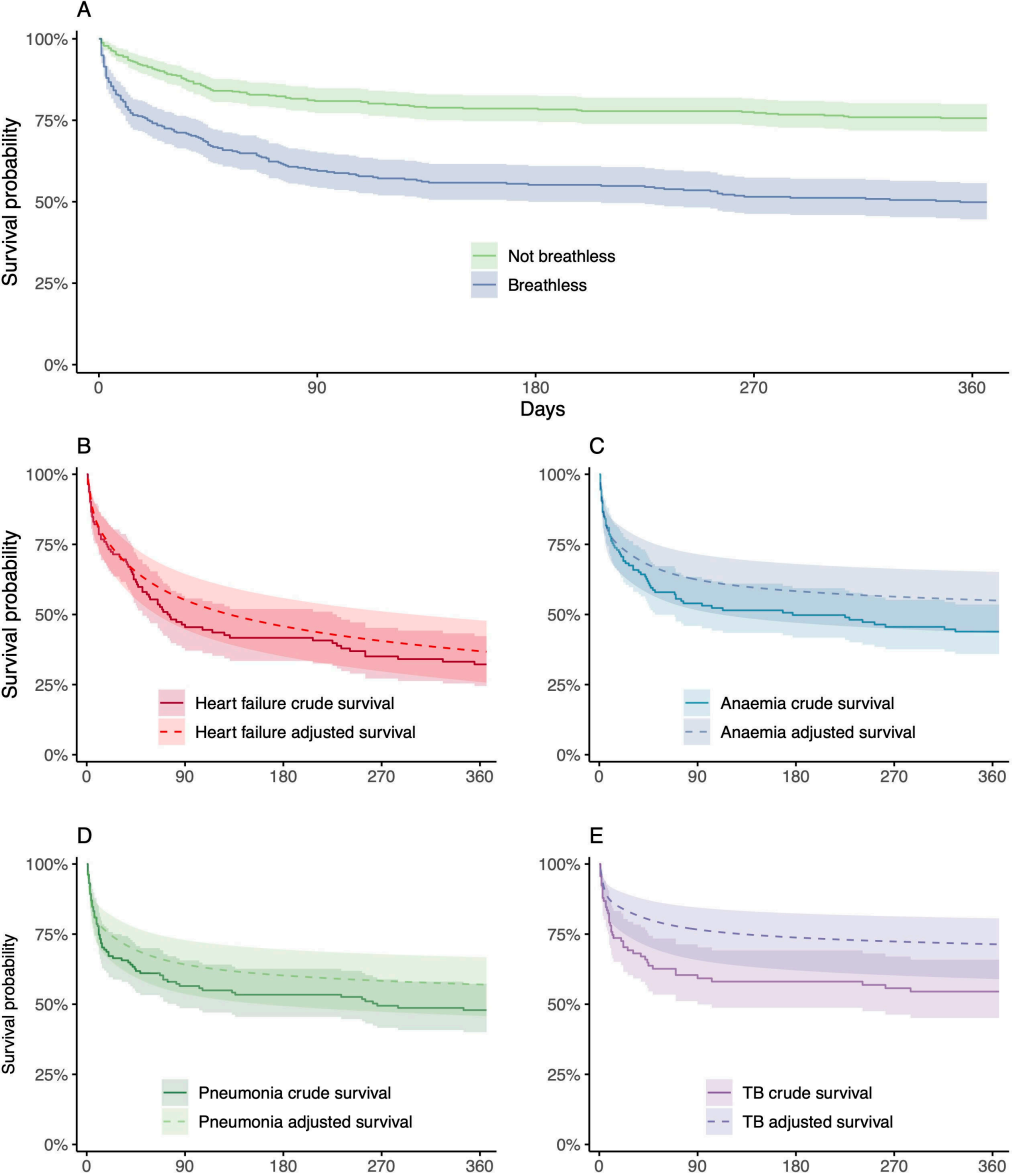
Survival plots. (**A–E**) Solid lines represent Kaplan-Meier survival plots with 95% CIs. (**B–E**) Dashed lines show survival (with 95% CIs) predicted from flexible parametric models, adjusted for the presence of conditions shown in figure 2. Full model details are available in online supplemental file section 6. Panel D reflects all-cause pneumonia (n=131) including TB-associated pneumonia (52/131 cases). A sensitivity analysis excluding cases of TB-associated pneumonia from panel D is presented in online supplemental figure S7, which demonstrates consistent findings.

Participant survival to hospital discharge, and then 30, 90 and 365 days after admission, is shown in table 4. Participants with breathlessness suffered significantly higher 1-year mortality (51% (157/307)) compared with those acute hospital admissions recruited to the MultiLink cohort who were not breathless (26% (100/385)); adjusted HR 1.8 (95% CI 1.4 to 2.3); figure 3; online supplemental table S27). Among participants with breathlessness, those with heart failure had the highest 1-year mortality (69% (59% to 77%); 75/109), followed by anaemia (57% (48% to 66%); 70/122), pneumonia (53% (44% to 61%); 68/129) and TB (47% (37% to 57%); 41/87). Figure 3 demonstrates unadjusted and adjusted survival (accounting for concurrent diagnoses of heart failure, anaemia, pneumonia and TB). This analysis demonstrates reduced mortality after adjustment for individual diseases for TB (29% (19% to 41%)), anaemia (45% (35% to 57%)) and pneumonia (43% (33% to 54%)), but not for heart failure (63% (52% to 74%)) compared with unadjusted survival (figure 3; online supplemental table S28). In our adjusted model, patients who died were more likely to have heart failure (HR 1.6 (95% CI 1.2 to 2.3)), higher admission UVA scores (HR 1.2 (1.1 to 1.3)) and were older (HR 1.02 (1.01 to 1.03); online supplemental table S32). Kaplan-Meier plots for all aetiologies are presented in online supplemental figure S6.

**Table 4:** Outcomes

	Hospital	Day 30	Day 90	1 Year
Mortality	72/316 (23%)	88/316 (28%)	128/316 (41%)	157/307 (51%)
Heart failure	25/112 (22%)	33/112 (29%)	61/112 (54%)	75/109 (69%)
Anaemia	34/126 (27%)	41/126 (33%)	58/126 (46%)	70/122 (57%)
Pneumonia	38/131 (29%)	44/131 (34%)	57/131 (44%)	68/129 (53%)
Tuberculosis	24/91 (26%)	28/91 (31%)	37/91 (41%)	41/87 (47%)
COPD	1/21 (5%)	1/21 (5%)	3/21 (14%)	5/21 (24%)
Asthma	0/18 (0%)	0/18 (0%)	1/18 (6%)	3/17 (18%)
Acute myocardial infarction	2/8 (25%)	2/8 (25%)	3/8 (38%)	5/8 (63%)
Readmission (survivors)*	.	.	27/178† (15%)	40/149† (27%)
Hospital LOS				
LOS (all participants), median days (IQR)	6 (3–9)	.	.	.
LOS (survivors), median days (IQR)	6 (4–9)	.	.	.
mMRC				
Median (IQR)	2 (1–4)	.	.	0 (0–1)
Grade 0	75/316 (24%)	.	.	94/150 (63%)
Grade 1	69/316 (22%)	.	.	35/150 (23%)
Grade 2	67/316 (21%)	.	.	19/150 (13%)
Grade 3	21/316 (7%)	.	.	0/150 (0%)
Grade 4	84/316 (27%)	.	.	2/150 (1%)
Disability‡				
With	170/316 (54%)	.	16/168† (10%)	.
Without	146/316 (46%)	.	152/168† (90%)	.
EQ5D-5L				
HRQoL health utility score, median (IQR)	0.52 (0.23–0.66)	0.94 (0.68–1.00)	0.94 (0.76–1.00)†	0.94 (0.73–11.00)
EQ5D-VAS, median (SD)	52.7 (15.7)	76.0 (18.7)	80.1 (18.1)†	80.2 (16.1)

* Number of participants who survived to follow-up and had at least one hospital admission between the index admission and follow-up.

† Disability and EQ5D-5L data were available for 168 survivors at the 90-day follow-up. Readmission data were available for 178 survivors at the 90-day follow-up, and 149 at the 1-year follow-up.

‡ Disability determined by Washington Group-Short Set on Functioning.^21^

COPD, chronic obstructive pulmonary disease; EQ5D VAS, EQ5D-visual analogue score; HRQoL, health-related quality of life; LOS, length of stay; mMRC, modified Medical Research Council.

Patient-reported outcome measures and functional outcomes are reported in table 4 (further details and condition-specific results in online supplemental tables S33–S37 and figure S8). Patients who died had higher admission mMRC (HR 1.2 (95% CI 1.0 to 1.3) per point increase); clinical frailty scale (HR 1.3 (1.2 to 1.5) per point increase) and disability (HR 2.3 (1.6 to 3.2); online supplemental table S31). A longer duration between symptom onset and hospital presentation was statistically associated with increased mortality (HR 1.04 (1.01 to 1.07) per week). HIV infection was not associated with mortality (participants with HIV 47% (53/114) 1-year mortality; participants without HIV 53% (100/189); HR 0.9 [0.7 to 1.3)). Mortality rates for each aetiology, disaggregated by HIV status, are presented in the online supplemental table S30.

**Figure 4:**
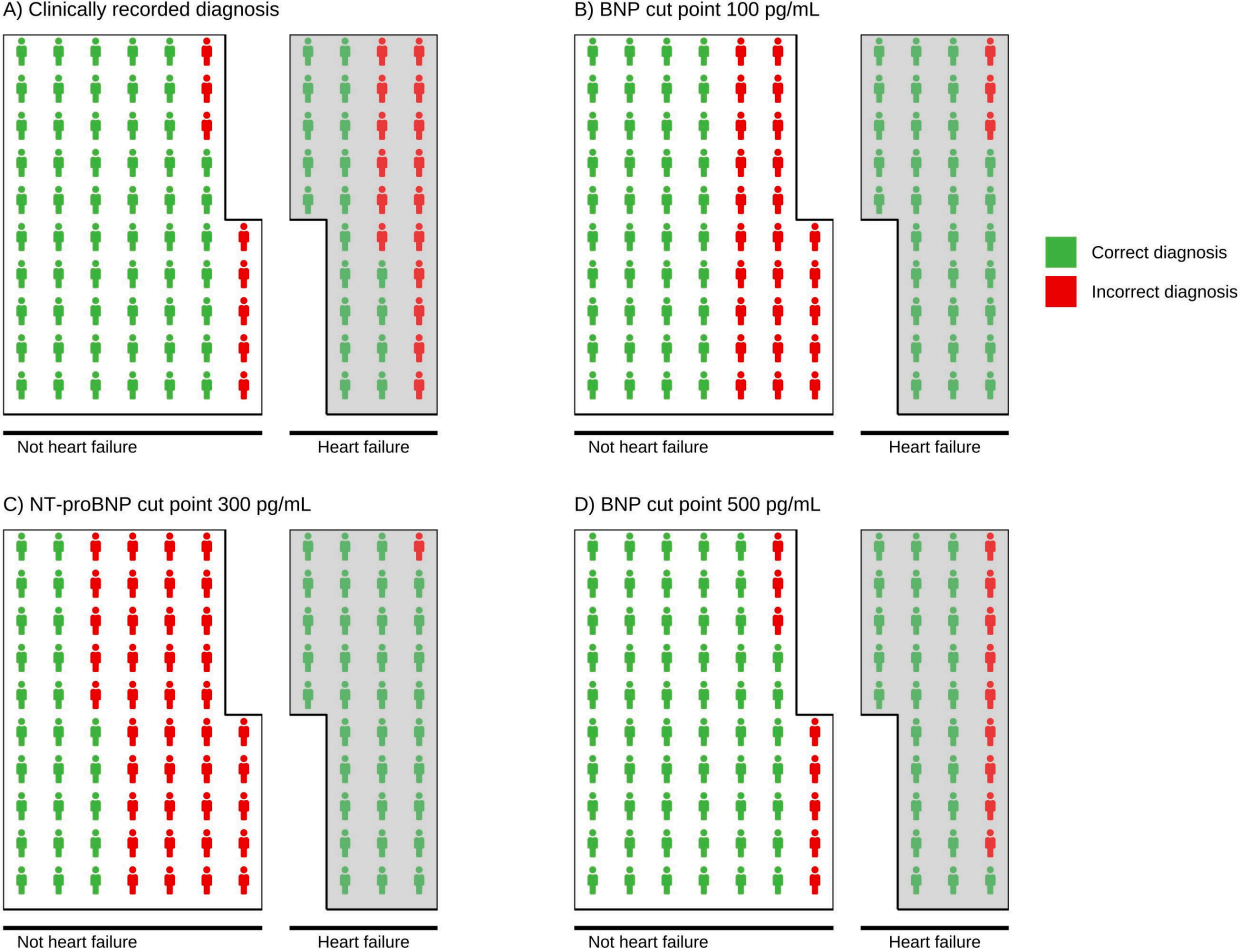
Diagnostic accuracy. Each plot represents 100 participants (1 icon=1%), illustrating a 35% prevalence of heart failure diagnosis (reference standard positive, determined by the Universal Definition of Heart failure^22^; shaded grey) in our cohort. The plots depict the proportion of correct and incorrect diagnoses, including true positives and false negatives (within the shaded sections), as well as true negatives and false positives (in the unshaded sections). (**A**) Clinically recorded diagnosis refers to hospital clinician-reported diagnosis in medical notes (ie, current standard of care); (**B**) BNP threshold: 100 pg/mL; (**C**) NT-proBNP threshold: 300 pg/mL; (**D**) BNP threshold: 500 pg/mL. The BNP 100 pg/mL and NT-proBNP 300 pg/mL thresholds are internationally recognised rule-out levels^22^ and the higher BNP threshold of 500 pg/mL, identified to enhance specificity (online supplemental tables S38 and S39 for further details). BNP, brain natriuretic peptide; NT-proBNP, N-terminal pro-B-type natriuretic peptide.

We compared the diagnostic accuracy of BNP (AUC 0.89 (95% CI 0.85 to 0.93)) and NT-proBNP (AUC 0.88 (95% CI 0.84 to 0.92)) for prediction of heart failure with our gold standard diagnosis (universal definition^22^). Overall, we showed sensitivity of BNP at 100 pg/mL and NT-proBNP at 300 pg/mL at 92.7% (95% CI 86.2 to 96.8) and 98.2% (93.5 to 99.8), respectively, compared with the standard of care (54.0% (44.8 to 63.9)). The specificities for BNP at 100 pg/mL and NT-proBNP at 300 pg/mL were 60.8% (53.7 to 67.6) and 38.1% (31.3 to 45.2), respectively, when compared with standard of care (88.1% (82.8 to 92.2)). Figure 4 displays the diagnostic accuracy data for clinical determination of heart failure (blinded to echocardiography and serum BNP/NT-proBNP) and established serum BNP and NT-proBNP thresholds from high-income settings. We also evaluated the diagnostic accuracy of CRP (AUC 0.77 (0.72 to 0.82)) and PCT (AUC 0.69 (0.63 to 0.75)) for diagnosis of pneumonia. The sensitivity of CRP at >25 mg/L and PCT at >0.25 ng/mL was 86.8% (79.7 to 92.1) and 72.1% (63.5 to 79.6), respectively. The specificities of CRP at >25 mg/L and PCT at >0.25 ng/mL were 46.9% (39.4 to 54.5) and 54.2% (45.7 to 61.0), respectively. In our decision curve analyses, across most threshold probabilities for treating heart failure, there was a net benefit to using BNP positivity at 100 pg/mL as the basis for treatment (see online supplemental figure S10). However, no net benefit was observed for CRP positivity at 25 mg/L or PCT positivity at 0.25 ng/mL in guiding treatment for pneumonia (see online supplemental figures S11 and S12). AUC curves and additional diagnostic accuracy indices are provided in online supplemental figures S9–S12 and tables S38 and S39.

## Discussion

In our multicentre, prospective cohort study in Malawi, we found that breathlessness was a common cause of medical admission with poor outcome—over 50% of breathless patients died within a year of admission. Pneumonia, anaemia, heart failure and TB were the most common diagnoses. Nearly two-thirds of participants had multiple conditions, a finding associated with increased mortality. This is important because current models of care in sub-Saharan Africa are focused on individual, predominantly infective, diseases. We recommend interventional studies with clinical and economic evaluation to determine if integrated approaches to diagnose and treat breathlessness can improve survival in this high-risk patient group and support effective and efficient healthcare resource allocation.

The average age of our cohort with breathlessness was 52 years. While this is similar to other medical admission cohorts in Africa (often between 37 and 53 years),^24^ but approximately 16 years younger than comparable cohorts from high-income settings (68; 51–80).^25^ Despite this younger age, we observed a higher proportion of heart failure (35%) among our participants compared with people admitted with dyspnoea in high-income countries (17%).^25^ This likely reflects the rising burden of non-communicable diseases (such as hypertension) and high prevalence of untreated valve disease in sub-Saharan Africa. These are often underdiagnosed, undercontrolled^24^ and lead to end-organ complications, as demonstrated by the high proportion of hypertensive heart disease in our cohort. The high prevalence of pneumonia and TB aligns with regional data^26 27^ and likely reflects the high HIV infection prevalence (identified in 50%–60% of participants with pneumonia and TB in our cohort). Similarly, the burden of anaemia has been well-documented in sub-Saharan Africa, where multifactorial aetiology has been described^28^ (although we were unable to delineate precise causes in our study). We identified a relatively low prevalence of both COPD (7%) and MI (3%) in our study. All cases of MI were type 2 (secondary to a mismatch in myocardial oxygen supply and demand), with no cases of type 1 MI (secondary to atherosclerotic disease) detected. In contrast, the Global Burden of Disease (GBD) estimates have predicted a much higher burden from both COPD and MI, both at a regional and global level, compared with the other conditions captured in our study, such as TB, lower respiratory tract infections and hypertensive heart disease.^29^ Our results align well with empirical observations from the region,^24^ and these data may therefore indicate a lower burden of COPD and MI than the modelled GBD estimates (which include limited empirical data from sub-Saharan Africa) suggest.^29^

Mortality rates for heart failure, pneumonia, TB and anaemia in our study align with regional data that indicate substantially higher age-adjusted mortality rates than in high-income countries.^26 27 29^ For example, heart failure-related mortality in our cohort (69% at 1 year) was similar to previous observations from Africa (~60%),^30^ but nearly three times higher than European cohorts (23.6%),^31^ despite patients being on average 11 years younger (58 years vs 69 years).^31^ Similarly, pneumonia-related mortality in our study (52% at 1 year) aligns with empirical regional observations^32^ and is 50% higher than reports from the USA (31%), despite patients being on average 20 years younger (48 years vs 68 years).^33^ These disparities in survival are likely due to multiple factors. For example, limited diagnostic capacity impedes prompt initiation of effective treatment^4 24^; an issue compounded by complex clinical presentations and multiple concurrent pathologies. Furthermore, prehospital admission delays were common and statistically associated with mortality, but the clinical relevance is unclear from our dataset. Finally, postdischarge factors are likely to influence outcomes. For instance, patients with TB (and indeed HIV; online supplemental table S29) had higher survival after discharge, likely due to their access to relatively well-resourced, vertically delivered outpatient programmes, demonstrated to reduce TB mortality by 60%–70%.^34^ However, postdischarge mortality was substantial among participants with heart failure who suffered from the highest 1-year mortality risk. High-income countries have established care pathways that use non-physician cadres to deliver guideline-directed therapy for advanced non-communicable diseases like heart failure, but such systems are uncommon in sub-Saharan Africa. Linkage to enhanced postdischarge care will be critical to improve outcomes for patients with poor prognoses. We found that functional status indices (mMRC, clinical frailty score, disability) were strongly associated with mortality. Future research should assess their utility to identify high-risk patients for targeted interventions.

Improved diagnostic strategies are essential in LMICs, given the high prevalence of multiple contributing conditions. We found that single admission measurements of CRP and PCT did not accurately identify cases of pneumonia. This may reflect high levels of inflammation and critical illness associated with other pathologies within this cohort. In high-income settings, serial measurements have demonstrated utility in guiding antibiotic treatment decisions,^23^ but this approach may not currently be feasible in LMICs due to resource and financial constraints. While our study evaluated biomarkers not previously used for pneumonia diagnoses in the Malawi adult population, future research should also evaluate whether the addition of routinely available tests (such as white cell counts) to CRP/PCT could improve diagnostic accuracy. For heart failure diagnoses, we found that natriuretic peptide assays may be useful to rule out but not rule in diagnoses. This is consistent with data from high-income countries, where natriuretic peptides are used to identify patients who require echocardiographic diagnostic confirmation.^22^ Further studies should evaluate heart failure diagnostic strategies in low-resource settings (echocardiography and cardiology expertise are not routinely available) before BNP can be recommended in diagnostic guidelines. This may involve use of higher cut-points for natriuretic peptides that have higher specificity, or through development of diagnostic algorithms that integrate other readily available clinical information—such as symptoms, clinical signs or ultrasound, increasingly available in LMIC hospitals, to improve diagnostic accuracy. Diagnostic strategies should be co-developed with key stakeholders including patients, healthcare workers and policy makers, including multidisciplinary clinical, health-economic and health-systems perspectives.

To our knowledge, this is the first study to systematically evaluate diagnoses and outcomes associated with breathlessness in sub-Saharan Africa. Our definition of breathlessness was designed to be inclusive of both symptoms and physiological parameters, cognisant of pulse oximeter imprecision in darker skin tones when this marker is used in isolation.^11^ We deployed a systematic diagnostic approach, based on internationally accepted case diagnostic criteria, informed from a systematic search of diagnostic literature, inclusive of rigorous QA and QC, and supported by consultant cardiologists, radiologists and respiratory physicians with clinical expertise of healthcare delivery in Malawi.^13^ We followed up participants for 1 year, with minimal loss to follow-up. Resource constraints limited the diagnosis of pulmonary embolism, pulmonary hypertension, interstitial lung disease and malignancy. Spirometry for COPD diagnoses was conducted at day 90 (to allow for recovery from acute illness) and therefore susceptible to survivorship bias. We were not able to differentiate between new-onset acute pathology and acute-on-chronic presentations in our study due to limited access to diagnostics in Malawi. The majority of our diagnostic criteria were derived from high-income settings (such as cardiac dimensions),^13^ which highlights the need for locally validated tools to improve diagnostic precision. Our study would have been strengthened by wider inclusion of patients from other countries, but this was not possible within the logistical and financial constraints of this study.

In summary, our multicentre prospective cohort study in Malawi demonstrates that breathlessness is a common reason for hospital admission, frequently involves multiple concurrent conditions and is associated with high morbidity and mortality. Interventional studies are needed to determine if integrated acute-care pathways with context-sensitive diagnostic and treatment approaches can sustainably and cost-effectively improve hospital survival. After successful hospital discharge, research is required to determine if community care interventions can improve long-term outcomes.

## Supplementary material

10.1136/thorax-2025-223623 online supplemental file 1

10.1136/thorax-2025-223623 online supplemental file 2

10.1136/thorax-2025-223623 online supplemental file 3

## Data Availability

Data are available on reasonable request.
