# Supplementary material for: Acute breathlessness as a cause of hospitalisation in Malawi: a prospective, patient-centred study to evaluate causes and outcomes
Source: Thorax. 2025 Sep 10;81(4):e223623. doi: 10.1136/thorax-2025-223623 (PMC13018744; doi:10.1136/thorax-2025-223623)
Supplement: online supplemental file 1 [file thorax-81-4-s001.pdf]

Table of Contents

Guide to supplemental material 3

Section 1. Checklists 4
Table S 1. STROBE Checklist 4
Table S 2. STARD Checklist 5

Section 2. Case definitions and diagnostic pathways 6
Pneumonia diagnostic criteria 6
Table S 3. Pneumonia diagnosis 6
Tuberculosis diagnostic criteria 7
Table S 4. TB: microbiological diagnosis 7
Table S 5. TB: radiological or clinical diagnosis 7
Table S 6. TB: microbiological or clinical or radiological criteria. 7
Post-TB lung disease 7
Heart failure diagnostic criteria 8
Echocardiography assessment: further details 8
Table S 7. Heart failure diagnosis 8
Table S 8. Echocardiographic assessment of ejection fraction (EF) 9
Myocardial infarction diagnostic criteria 9
Table S 9. Acute myocardial infarction diagnosis 9
COPD diagnostic criteria 10
Table S 10. COPD diagnosis 10
Asthma diagnostic criteria 10
Table S 11. Asthma diagnosis 10
Anaemia diagnostic criteria 11
Table S 12. Anaemia diagnosis 11
Pulmonary embolism diagnostic criteria 12
Table S 13. Pulmonary embolism diagnosis 12
Pulmonary hypertension diagnostic criteria 13
Table S 14. Pulmonary hypertension diagnosis 13
Pneumothorax diagnostic criteria 14
Table S 15. Pneumothorax diagnosis 14
Pleural effusion diagnostic criteria 15
Table S 16. Pleural effusion diagnosis 15
Table S 17. Diagnoses through consensus 15
Figure S 1 Pairwise disease prevalence 16

Section 3. Components of breathlessness definition 17
Figure S 2. Euler plot. Overlap between "shortness of breath", hypoxaemia and tachypnoea among enrolled participants. 17
Table S 18. Mortality statistics for patients with hypoxaemia, tachypnoea and "shortness of breath". 17

Section 4. Disease sub-classification 18
Table S 19. Pneumonia aetiology and TB sub-classification 18
Figure S 3. Euler plot showing overlap between cases of pneumonia and TB 19
Table S 20. Echocardiographic evidence of cardiac abnormalities 20
Table S 21. Pulmonary hypertension diagnoses by echocardiography 22
Table S 22. Cardiac arrhythmia 22
Table S 23. Myocardial infarction 22
Table S 24. Obstructive lung disease 23
Table S 25. Haematological and vascular causes of breathlessness 23
Table S 26. Pleural disease 24
Figure S 4. Euler plot showing overlap between pleural effusion with heart failure, pneumonia and TB. 24

Section 5. Outcomes: whole cohort (Multilink) 25
Table S 27. Breathlessness and other risk factors for mortality among the entire medical cohort. 25

Section 6. Supplementary statistical methods: model development 26
Flexible parametric model: statistical details 26
Survival predictions based on the flexible parametric model 26

Figure S 5. Flexible parametric model fit ... 27

Section 7. Outcomes: breathless cohort ... 28

Table S 28. Unadjusted and adjusted 30-day, 90-day and one-year mortality for heart failure, anaemia, pneumonia and TB: results from flexible parametric model ... 28
Figure S 6. Kaplan Meier survival plots for all aetiologies ... 29
Figure S 7. Survival plot sensitivity analysis ... 29
Table S 29. In-hospital, 30-day, 90-day and 1-year mortality, stratified by demographic factors, HIV infection status, functional measures, and diagnoses ... 30
Table S 30. HIV status and one-year mortality by diagnosis. ... 31
Table S 31. Association between functional and demographic factors and mortality. Univariable flexible parametric models ... 32
Table S 32. Association between UVA, age, sex, heart failure, anaemia, pneumonia, TB and mortality: Flexible parametric model ... 32
Table S 33. EQ5D-5L results by category at each follow-up ... 33
Figure S 8. Distribution of HRQoL health utility scores among survivors at each follow-up ... 34
Table S 34. Demographic details, functional and clinical outcomes among participants with heart failure ... 35
Table S 35. Demographic details, functional and clinical outcomes among participants with anaemia ... 36
Table S 36. Demographic details, functional and clinical outcomes among participants with pneumonia ... 37
Table S 37. Demographic details, functional and clinical outcomes among participants with TB ... 38

Section 8. Diagnostic accuracy supplemental data ... 39

Figure S 9. Diagnostic accuracy of biomarkers for heart failure and pneumonia: receiver operator curves ... 39
Table S 38. Diagnostic accuracy of natriuretic peptides for heart failure diagnosis ... 40
Table S 39. Diagnostic accuracy of C-reactive peptide and procalcitonin for pneumonia diagnosis ... 40
Figure S 10. Decision curve analysis of BNP (cut point 100 pg/mL) for heart failure ... 41
Figure S 11. Decision curve analysis of CRP (cut point 25 mg/l) for pneumonia ... 41
Figure S 12. Decision curve analysis of PCT (cut point 0.25ng/ml) for pneumonia ... 41

References ... 42

Guide to supplemental material

Section 1. Checklists
This section presents the strobe and STARD checklists

Section 2. Case definitions and diagnostic pathways.
This section presents the case definitions for each aetiology screened. The diagnostic pathways describe how many participants were diagnosed according to different diagnostic techniques incorporated within each case definition. These tables also provide details, and the number of participants classified, when substitute case definitions were applied or panel (consensus) approaches were utilised in instances of incomplete diagnostic data.

Section 3. Components of the breathlessness definition.
This section presents the overlap between each constituent symptom and sign for our breathlessness conditions. We also present mortality disaggregated by each of these components.

Section 4. Disease sub-classification.
Here, we provide additional data on the aetiology of each cause of breathlessness we screened for.

Section 5. Outcomes: whole cohort.
This section includes a table of risk factors for mortality among the whole cohort and demonstrates the impact of the breathlessness syndrome on mortality rate.

Section 6. Supplementary statistical methods: model development.
This section includes a description of our model building strategy for the included survival analyses.

Section 7. Outcomes: breathlessness cohort
This section includes supplementary data on patient outcomes (survival, functional and health-related quality of life), stratified by aetiology.

Section 8. Diagnostic accuracy supplemental data
This section presents the receiver operator curves, decision curve analyses and the results of additional diagnostic accuracy indices and exploratory cut-points.

Section 1. Checklists

Table S 1. STROBE Checklist

	Item No	Recommendation	Location in manuscript
**Title and abstract**	1	(*a*) Indicate the study's design with a commonly used term in the title or the abstract	pp 1,3
		(*b*) Provide in the abstract an informative and balanced summary of what was done and what was found	pp 3-4
**Introduction**			
Background/rationale	2	Explain the scientific background and rationale for the investigation being reported	pp 6-8
Objectives	3	State specific objectives, including any prespecified hypotheses	p 7
**Methods**			
Study design	4	Present key elements of study design early in the paper	p 8; Published protocol (doi.org/10.12688/wellcomeopenres.21041.1)
Setting	5	Describe the setting, locations, and relevant dates, including periods of recruitment, exposure, follow-up, and data collection	p 8; Published protocol (doi.org/10.12688/wellcomeopenres.21041.1)
Participants	6	Give the eligibility criteria, and the sources and methods of selection of participants. Describe methods of follow-up	p 9; Published protocol (doi.org/10.12688/wellcomeopenres.21041.1)
Variables	7	Clearly define all outcomes, exposures, predictors, potential confounders, and effect modifiers. Give diagnostic criteria, if applicable	pp 9-11; Published protocol (doi.org/10.12688/wellcomeopenres.21041.1); Appendix pp 3-15
Data sources/ measurement	8	For each variable of interest, give sources of data and details of methods of assessment (measurement). Describe comparability of assessment methods if there is more than one group	pp 9-12; Published protocol (doi.org/10.12688/wellcomeopenres.21041.1)
Bias	9	Describe any efforts to address potential sources of bias	Published protocol (doi.org/10.12688/wellcomeopenres.21041.1)
Study size	10	Explain how the study size was arrived at	p 13
Quantitative variables	11	Explain how quantitative variables were handled in the analyses. If applicable, describe which groupings were chosen and why	pp 11, 13
Statistical methods	12	(*a*) Describe all statistical methods, including those used to control for confounding	pp 13-14
		(*b*) Describe any methods used to examine subgroups and interactions	pp 13-14
		(*c*) Explain how missing data were addressed	pp 13-14
		(*d*) If applicable, explain how loss to follow-up was addressed	pp 13-14
		(*e*) Describe any sensitivity analyses	pp 13-14, 15, 36
**Results**			
Participants	13*	(a) Report numbers of individuals at each stage of study—eg numbers potentially eligible, examined for eligibility, confirmed eligible, included in the study, completing follow-up, and analysed	Figure 1
		(b) Give reasons for non-participation at each stage	Figure 1
		(c) Consider use of a flow diagram	Figure 1
Descriptive data	14*	(a) Give characteristics of study participants (eg demographic, clinical, social) and information on exposures and potential confounders	Table 1
		(b) Indicate number of participants with missing data for each variable of interest	Table 1; Tables S1-S24
		(c) Summarise follow-up time (eg, average and total amount)	pp 15; Figure 3
Outcome data	15*	Report numbers of outcome events or summary measures over time	pp 15-19; Figure 3
Main results	16	(*a*) Give unadjusted estimates and, if applicable, confounder-adjusted estimates and their precision (eg, 95% confidence interval). Make clear which confounders were adjusted for and why they were included	pp 15-19; Figures 2-3; Tables 2-3; Tables S15-S40; Fig S1-S6,
		(*b*) Report category boundaries when continuous variables were categorized	Table 1; S15-S40; Fig S1-S6,
		(*c*) If relevant, consider translating estimates of relative risk into absolute risk for a meaningful time period	pp 17-18
Other analyses	17	Report other analyses done—eg analyses of subgroups and interactions, and sensitivity analyses	Tables S1-S42; Figures S1-S12
**Discussion**			
Key results	18	Summarise key results with reference to study objectives	p 20
Limitations	19	Discuss limitations of the study, taking into account sources of potential bias or imprecision. Discuss both direction and magnitude of any potential bias	pp 23-24
Interpretation	20	Give a cautious overall interpretation of results considering objectives, limitations, multiplicity of analyses, results from similar studies, and other relevant evidence	pp 20-25
Generalisability	21	Discuss the generalisability (external validity) of the study results	pp 20-25
**Other information**			
Funding	22	Give the source of funding and the role of the funders for the present study and, if applicable, for the original study on which the present article is based	p 27

Table S 2. STARD Checklist

Section & topic	No	Item	Location in manuscript
**Title or abstract**	**1**	Identification as a study of diagnostic accuracy using at least one measure of accuracy (such as sensitivity, specificity, predictive values, or AUC)	pp 3-4
**Abstract**	**2**	Structured summary of study design, methods, results, and conclusions (for specific guidance, see STARD for Abstracts)	pp 3-4
**Introduction**	**3**	Scientific and clinical background, including the intended use and clinical role of the index test	pp 6-7
	**4**	Study objectives and hypotheses	pp 7-8
**Methods**			
Study design	**5**	Whether data collection was planned before the index test and reference standard were performed (prospective study) or after (retrospective study)	pp 12. ; Published protocol (doi.org/10.12688/wellcomeopenres.21041.1)
Participants	**6**	Eligibility criteria	p 9; Published protocol (doi.org/10.12688/wellcomeopenres.21041.1)
	**7**	On what basis potentially eligible participants were identified (such as symptoms, results from previous tests, inclusion in registry)	p 9; Published protocol (doi.org/10.12688/wellcomeopenres.21041.1)
	**8**	Where and when potentially eligible participants were identified (setting, location and dates)	p 9; Published protocol (doi.org/10.12688/wellcomeopenres.21041.1)
	**9**	Whether participants formed a consecutive, random or convenience series	p 9
Test methods	**10a**	Index test, in sufficient detail to allow replication	p 12; Published protocol (doi.org/10.12688/wellcomeopenres.21041.1)
	**10b**	Reference standard, in sufficient detail to allow replication	10; Published protocol (doi.org/10.12688/wellcomeopenres.21041.1)
	**11**	Rationale for choosing the reference standard (if alternatives exist)	pp 10-11; Published protocol (doi.org/10.12688/wellcomeopenres.21041.1)
	**12a**	Definition of and rationale for test positivity cut-offs or result categories of the index test, distinguishing pre-specified from exploratory	p 12; Published protocol (doi.org/10.12688/wellcomeopenres.21041.1)
	**12b**	Definition of and rationale for test positivity cut-offs or result categories of the reference standard, distinguishing pre-specified from exploratory	p 12; Published protocol (doi.org/10.12688/wellcomeopenres.21041.1)
	**13a**	Whether clinical information and reference standard results were available to the performers/readers of the index test	p 12
	**13b**	Whether clinical information and index test results were available to the assessors of the reference standard	p 12
Analysis	**14**	Methods for estimating or comparing measures of diagnostic accuracy	p 13; Published protocol (doi.org/10.12688/wellcomeopenres.21041.1)
	**15**	How indeterminate index test or reference standard results were handled	p 13
	**16**	How missing data on the index test and reference standard were handled	p 13
	**17**	Any analyses of variability in diagnostic accuracy, distinguishing pre-specified from exploratory	NA
	**18**	Intended sample size and how it was determined	p 13
**Results**			
Participants	**19**	Flow of participants, using a diagram	Figure 1
	**20**	Baseline demographic and clinical characteristics of participants	Table 1
	**21a**	Distribution of severity of disease in those with the target condition	Tables S38-S40
	**21b**	Distribution of alternative diagnoses in those without the target condition	Figure 2
	**22**	Time interval and any clinical interventions between index test and reference standard	pp 10-13
Test results	**23**	Cross tabulation of the index test results (or their distribution) by the results of the reference standard	Figure 4, Tables S42-43
	**24**	Estimates of diagnostic accuracy and their precision (such as 95% confidence intervals)	Figure S9-12; Tables S42-43
	**25**	Any adverse events from performing the index test or the reference standard	NA
**Discussion**	**26**	Study limitations, including sources of potential bias, statistical uncertainty, and generalisability	pp 23-24
	**27**	Implications for practice, including the intended use and clinical role of the index test	pp 22-23
**Other information**	**28**	Registration number and name of registry	NA
	**29**	Where the full study protocol can be accessed	Published protocol (doi.org/10.12688/wellcomeopenres.21041.1)
	**30**	Sources of funding and other support; role of funders	pp 27

Section 2. Case definitions and diagnostic pathways

Pneumonia diagnostic criteria

Pneumonia diagnoses were based on the Infectious Diseases Society of America/American Thoracic Society (ATS) Consensus Guidelines[1]:

- "Clinical features (cough, fever, sputum production, and pleuritic chest pain) supported by a demonstrable infiltrate by chest radiograph or other imaging technique, with or without supporting microbiological data, is required for the diagnosis of pneumonia".

Table S 3. Pneumonia diagnosis

	Diagnostic step	n, positive	n, negative	unclassified
**1. IDSA / ATS criteria with both chest x-ray (CXR) and LUS data available**	1. Positive or negative	130	164	22
**2. IDSA / ATS criteria: either LUS or CXR information available.**	2. Negative if no evidence of consolidation on one imaging modality and the other modality missing or unclear	130	183	3
**3. Panel consensus diagnosis**		131	185	0
	**Total, n (%)**	131 (41%)	185 (59%)	0 (0%)

Tuberculosis diagnostic criteria

Tuberculosis (TB) was defined as:

1) Microbiologically confirmed TB If any of the following are positive[2]:

- Xpert TB/RIF
- Urine LAM

2) Clinical / radiologically suspected TB. In the absence of any positive microbiological tests for MTB [3] :

- Clinical diagnosis of TB in the presence of either compatible clinical illness or radiological disease
- And/or decision to start TB treatment by the responsible clinical team.

Table S 4. TB: microbiological diagnosis

	Diagnostic step	n, positive	n, negative	unclassified
**1. Microbiological diagnosis**	1. Xpert	20	123	173
	2. Urine LAM	26	37	253
	3. Microbiological diagnosis: positive if either Xpert or LAM positive, negative if both Xpert and LAM negative	45	129	142
	**Total, n (%)**	45 (14%)	129 (41%)	142 (45%)

Table S 5. TB: radiological or clinical diagnosis

	Diagnostic step	n, positive	n, negative	unclassified
**1. Radiological diagnosis**	1. Radiological TB diagnosis	26	256	34
**2. Clinical diagnosis**	2. Clinical TB diagnosis	51	263	2
**3. Patient reported diagnosis with ongoing treatment prescription**	3. Patient reported diagnosis ongoing treatment prescription	10	306	0
**4. Anti-TB medications prescribed**	4. Anti-TB prescription	36	280	0
**Total: Radiological or clinical diagnosis and/or on treatment**	**Total, n (%)**	69 (22%)	247 (78%)	0 (0%)

Table S 6. TB: microbiological or clinical or radiological criteria.

	Diagnostic step	n, positive	n, negative	unclassified
**Microbiological criteria**	1. Xpert TB or Urine LAM	45	129	142
**Clinical criteria**	2. Radiological or clinical diagnosis or on treatment	69	247	0
**Either microbiological or clinical or radiological criteria**	**Total, n (%)**	91 (29%)	225 (71%)	0 (0%)

Post-TB lung disease

20/282 (7%)*

*A diagnosis of post TB lung disease was made by consensus review of chest radiographs. The panel consisted of a consultant radiologist and acute care physician.

Heart failure diagnostic criteria

Heart failure diagnostic criteria will be based on the Universal Definition of Heart failure[4]*

- Symptoms and/or signs of heart failure
- Evidence of a structural and/or functional cardiac abnormality, including at least one of:
  - EF <50%
  - abnormal cardiac chamber enlargement
  - E/E′ >15
  - Moderate/severe ventricular hypertrophy
  - Moderate/severe valvular stenosis or regurgitation.
- Objective evidence of cardiogenic or systemic congestion with available imaging:
  - Chest x-ray (CXR)
  - Lung ultrasound
  - Elevated filling pressures by echocardiography
- HF with reduced EF (HFrEF): HF with LVEF ≤40%
- HF with mildly reduced EF (HFmrEF): HF with LVEF 41–49%
- HF with preserved EF (HFpEF): HF with LVEF ≥50%.

Note: natriuretic peptides, as one of the index tests of the study, did not form part of our definition for heart failure.

Echocardiography assessment: further details

Reference intervals for cardiac dimensions were based on British Society of Echocardiography (BSE) guidelines[5] and the Normal Reference Ranges for Echocardiography (NORRE) study.[6]

Ejection fraction was primarily calculated by Simpson's biplane method. In instances where biplane measurements were not possible due to image quality, ejection fraction was based on a single apical four chamber (A4c) view, if possible, or qualitatively visually assessed and agreed on by consensus, in line with guidance.[7] We have reported the proportion of cases ascertained by each method below (Table S 6).

We assessed LV diastolic dysfunction and elevated filling pressure using algorithms in the most recent guidelines from the BSE[8]; this superseded the approach listed in our previously published protocol manuscript.[9]

Table S 7. Heart failure diagnosis

	Diagnostic step	n, positive	n, negative	unclassified
**1. Universal definition of heart failure**	Positive or negative based on universal definition of heart failure with all available parameters	108	187	21
**2. BSE diastolic guidelines**	If E/E' incomplete: follow BSE diastolic dysfunction guidelines.	108	189	19
**3. If CXR/LUS incomplete: AHA / ESC criteria**	If CXR / LUS incomplete –use echocardiographic parameters alone (in line with AHA / ESC criteria):<br>- HF present if EF <40%<br>- HF present if EF 40-49% and objective evidence of structural heart disease or functional alterations*:<br>- raised LV mass<br>- raised LA volume<br>- LV diastolic dysfunction or elevated filling pressures	110	194	12
**4. Panel consensus diagnosis**	D) Panel consensus diagnosis	112	204	0
**Total, n (%)**		112 (35%)	204 (65%)	0 (0%)

*Assessment based on British Society of Echocardiography recommended reference intervals for cardiac dimensions and diastolic dysfunction guidelines.[5,6,8]

Table S 8. Echocardiographic assessment of ejection fraction (EF)

	n (%) cases
**Biplane EF**	289/304 (95%)
**Single plane A4C EF**	3/304 (1%)
**Qualitative EF assessment: consensus agreement***	12/304 (4%)

A4c: Apical four chamber.
*Consensus agreement by two echocardiographers.

Myocardial infarction diagnostic criteria

Fourth Universal Definition of Myocardial Infarction[10]

- Evidence of a rise and/or fall of cTnI values (>20% variation) with at least one value above the 99th percentile, and at least 1 of the following:
    - Symptoms of myocardial ischemia
    - Ischemic ECG changes
    - Pathological Q waves
    - Echocardiographic evidence of a regional wall motion abnormality in a pattern consistent with an ischemic aetiology.

Table S 9. Acute myocardial infarction diagnosis

	Diagnostic step	n, positive	n, negative	unclassified
**1. Universal definition[10]**	Diagnosis per criteria in Fourth Universal Definition of MI	8	290	18
**2. Second troponin not available: criteria for T3MI definition in Universal Definition[10]**	2. Positive if first troponin raised, patient died before 2nd troponin and ECG suggestive of presumed new ischemic ECG changes,	8	290	18
**3. Cardiac biomarkers not available.**	3. Negative if no evidence of RWMA on echo. Positive if evidence of RWMA on echo and symptoms of myocardial ischaemia present.	8	305	3
**4. Cardiac biomarkers not available.***	4. Negative if no evidence of pathological Q waves. Positive if evidence of pathological Q waves and symptoms of myocardial ischaemia present.*	8	306	2
**5. Panel consensus diagnosis**	5. Panel consensus diagnosis	8	308	0
**Total, n (%)**	**Total, n (%)**	8 (3%)	308 (97%)	0 (0%)

*Universal Definition of MI[10] and WHO definition of MI in low resource settings[11]: “when data on cardiac biomarkers are not available or incomplete, the diagnosis of MI can be confirmed by the development of pathological Q waves”.

COPD diagnostic criteria

COPD was defined following ERS/ATS criteria[12,13]:

- FEV1 / FVC < 5th percentile (z-score < -1.645)
- No evidence of reversibility (post-bronchodilator change in FEV1 ≤10%)[12]
- Symptoms including chronic dyspnoea, chronic cough or sputum production

Table S 10. COPD diagnosis

	Diagnostic step	n, positive	n, negative	unclassified
**1. Gold standard diagnosis:**	ERS/ATS criteria	15	203	98
**2. Spirometry not conducted**	Positive if symptoms + radiological signs on CXR defined by consultant radiologist in keeping with COPD	21	286	9
**3. Panel consensus diagnosis**	Panel consensus diagnosis	21	295	0
**Total, n (%)**	**Total, n (%)**	21 (7%)	295 (93%)	0 (0%)

Asthma diagnostic criteria

Severe asthma exacerbation based on the ATS/ERS official definition for clinical trials[14]: "A hospitalisation because of asthma, requiring systemic corticosteroids"

Table S 11. Asthma diagnosis

	Diagnostic step	n, positive	n, negative	unclassified
**Primary outcome: ATS/ERS official definition**	ATS/ERS official definition	18	298	0
**Total, n (%)**		18 (6%)	298 (94%)	0 (0%)

Anaemia diagnostic criteria

WHO haemoglobin thresholds for the diagnosis of anaemia[15]

- Anaemia in non-pregnant women (age ≥15 years): <120g/l
- Anaemia in males (age ≥15 years): <130 g/l

Sub-classification by severity

Anaemia in non-pregnant women (age ≥15 years): < 120g/l

- Mild: 110-110 g/l
- Moderate: 80-109 g/l
- Severe: <80 g/l

Anaemia in males (age ≥15 years): < 130 g/l

- Mild: 110-129 g/l
- Moderate: 80-109 g/l
- Severe: <80 g/l

Haemoglobin levels were adjusted for altitude and smoking status per WHO guidelines[15]:

- by altitude >1000m per WHO guidance (- 2 g/l for participants in Blantyre, Chiradzulu and Hai Districts).
- by smoking status (smokers: -3 g/l; 1-2 packets/day -5g/l; ≥2packets/day -7g/l).

Table S 12. Anaemia diagnosis

	**Diagnostic step**	**n, positive**	**n, negative**	**unclassified**
**iSTAT Hb reading**	WHO definition	125	178	13
**Panel diagnosis including review of clinical diagnoses**	Panel consensus diagnosis	126	190	0
**Total, n (%)**	**Total, n (%)**	126 (40%)	190 (60%)	0 (0%)

Pulmonary embolism diagnostic criteria

We defined pulmonary embolism (PE) as:

- CT proven PE (limited availability and not a routine study procedure)
- Clinically suspected PE and treated with anticoagulants
- Echocardiographic diagnoses

In the absence of computed tomography (CT), echocardiographic criteria were followed to assess for PE.

- Confirmed PE (ERS/ESC guidelines[16]): visualised thrombi in the right heart or pulmonary artery (or main branches of the pulmonary artery) confirmed the diagnosis of PE.
- Suspected PE (ERS/ESC and BTS guidelines[16,17]):
  - High risk patient with haemodynamic instability (systolic BP < 90 mmHg, a drop of > 40 mmHg, or need for vasopressors to maintain systolic BP ≥90 mmHg).[16]
  - Echocardiographic findings of RV pressure overload (RV strain): RV dilation (basal RV/LV ratio >1.0); and/or McConnell's sign (reduced contractility of the RV free wall and normal / hyperkinetic RV apical contractility); and/or D-sign (flattening/deviation of the interventricular septum towards the LV).[16,18]
  - Absence of other clear causes of RV pressure overload.

Table S 13. Pulmonary embolism diagnosis

	**Diagnostic step**	**n, positive**	**n, negative**	unclassified
**1. CT diagnosis**	PE diagnosed by CTPA*	1	-	-
**2. Clinically suspected PE and treated with anticoagulants**	Clinical diagnosis	1	315	0
**2. Echocardiographic criteria**	Echocardiographic criteria	0	304	12
**3. Echocardiographic or CT or clinical diagnosis**	Echocardiographic or CT or clinical diagnosis	1	315	0
**Total, n (%)**	**Total, n (%)**	1 (0.3%)	315 (99.7%)	0 (0%)

*One participant in the had a CTPA (due to clinical suspicion) in the cohort study.

Pulmonary hypertension diagnostic criteria

We followed diagnostic protocols from the BSE on the assessment of PH.[19] PH was confirmed in cases of 'high probability of PH' following BSE criteria.[19] The full breakdown of low intermediate or high probability are provided in Table S 19.

Table S 14. Pulmonary hypertension diagnosis

	Diagnostic step	n, positive	n, negative	unclassified
**Echo criteria**	PH based on echocardiographic*	40	264	12
**Panel consensus diagnosis**	Panel consensus diagnosis**	41	275	0
**Total, n (%)**	**Total, n (%)**	41 (13%)	275 (87%)	0 (0%)

*Based on 'high probability of pulmonary hypertension' (the full breakdown is available in Table S 19).
**We identified one participant who had not had an echo, with radiographic (chest x-ray) evidence of RV dilatation, most likely secondary to cor pulmonale from interstitial pulmonary fibrosis and therefore considered to have pre-capillary pulmonary hypertension.

Pneumothorax diagnostic criteria

Pneumothorax was identified on chest radiographs as a visible visceral pleural line with air between it and the chest wall and absent pulmonary vessels[20].

Pneumothorax was identified sonographically where each of the following are fulfilled (in line with international consensus guidelines) [21] :

- Absence of lung sliding
- Absence of B-lines
- Either presence of a lung point or absence of a lung pulse.

Table S 15. Pneumothorax diagnosis

	Diagnostic step	n, positive	n, negative	unclassified
**CXR**	PTX diagnosed by CXR	3	279	34
**Lung ultrasound**	PTX diagnosed by lung ultrasound	0	302	14
**CXR or lung ultrasound**	PTX diagnosed by CXR or lung ultrasound	3	307	6
**Panel consensus diagnosis**	Panel consensus diagnosis	3	313	0
**Total, n (%)**	**Total, n (%)**	3 (1%)	313 (99%)	0 (0%)

Pleural effusion diagnostic criteria

Pleural effusion detection was by lung ultrasound and CXR. Pleural effusion was identified sonographically as an anechoic or hypoechoic fluid collection in the pleural space.[22]

Table S 16. Pleural effusion diagnosis

	Diagnostic step	n, positive	n, negative	unclassified
**1. CXR**	Pleural effusion diagnosed by CXR	67	214	35
**2. US**	Pleural effusion diagnosed by US	103	99	14
**3. CXR or US**	Pleural effusion diagnosed by CXR or US	118	192	6
**Panel consensus diagnosis**	Panel consensus diagnosis	118	198	0
**Total, n (%)**	Total, n (%)	118 (37%)	198 (63%)	0 (0%)

Table S 17. Diagnoses through consensus

Diagnosis	N (%)
Acute respiratory infection	13/316 (4%)
Seizure	5/316 (2%)
Sepsis	3/316 (1%)
Diabetic ketoacidosis	2/316 (1%)
Ascites	1/316 (0.3%)
Hypertensive crisis	1/316 (0.3%)
Meningitis	1/316 (0.3%)

Figure S 1 Pairwise disease prevalence

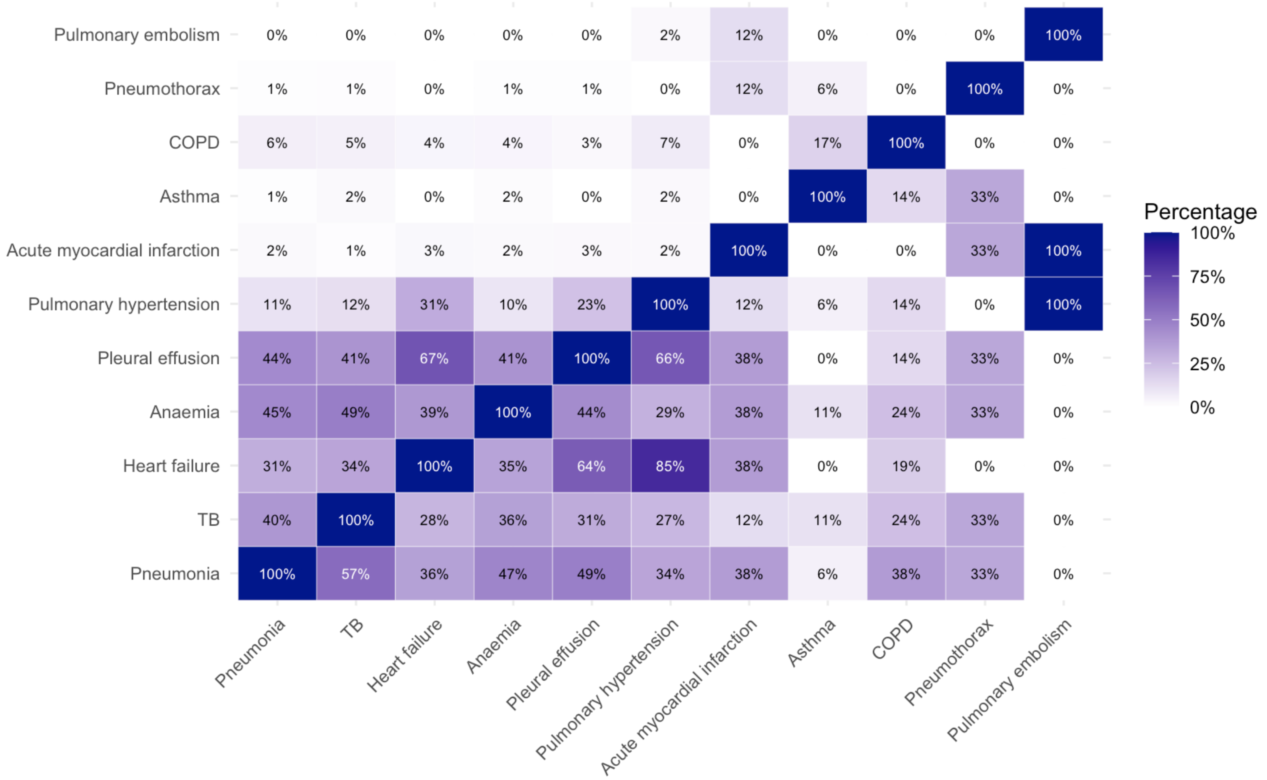

This figure presents pairwise prevalence between conditions with the primary condition in the x-axis; and concurrent conditions are within columns in the y axis. For example, the Figure Shows that 40% of participants with pneumonia also had TB; 57% of participants with TB also had pneumonia.

Section 3. Components of breathlessness definition

Figure S 2. Euler plot. Overlap between "shortness of breath", hypoxaemia and tachypnoea among enrolled participants.

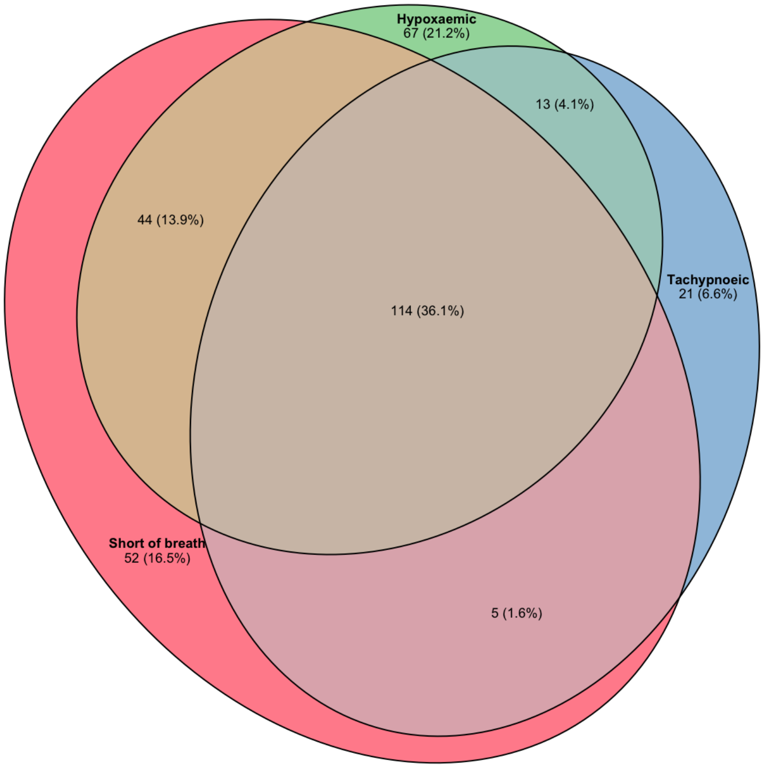

Hypoxaemia is defined as a composite of SpO2<94 and/or receiving oxygen therapy
Tachypnoea is defined as respiratory rate >25
Shortness of breath is defined as a participant complaining of "shortness of breath" as a symptom experienced within the week leading to admission.

Table S 18. Mortality statistics for patients with hypoxaemia, tachypnoea and "shortness of breath".

Variable	N	Hospital mortality, n (%) [95% CI]	D30 mortality, n (%) [95% CI]	D90 mortality, n (%) [95% CI]	1Y mortality, n (%) [95% CI]
Hypoxaemic*	176	52/176 (29.5%) [22.9-36.9]	58/176 (33%) [26.1-40.4]	79/176 (44.9%) [37.4-52.6]	96/174 (55.2%) [47.5-62.7]
Tachypnoeic	215	55/215 (25.6%) [19.9-32]	67/215 (31.2%) [25-37.8]	95/215 (44.2%) [37.4-51.1]	112/209 (53.6%) [46.6-60.5]
"Short of breath"	277	58/277 (20.9%) [16.3-26.2]	72/277 (26%) [20.9-31.6]	111/277 (40.1%) [34.3-46.1]	137/269 (50.9%) [44.8-57.1]

*Hypoxaemia was taken as a composite of SpO2<94 and/or receiving oxygen therapy

Section 4. Disease sub-classification

Table S 19. Pneumonia aetiology and TB sub-classification

	Chiradzulu	QECH	Total
**Pneumonia***	44/140 (31%)	87/176 (49%)	131/316 (41%)
Pneumonia aetiology			
Pneumonia, aetiology not defined	31/44 (70%)	64/87 (74%)	95/131 (73%)
Pneumonia with bacterial pathogen (excluding MTB)	0/44 (0%)	4/87 (5%)	4/131 (3%)
Blood culture results not available	9/44 (20%)	5/87 (6%)	14/131 (11%)
Pneumonia with viral pathogen	6/44 (14%)	5/87 (6%)	11/131 (8%)
Viral PCR not available	5/44 (11%)	4/87 (5%)	9/131 (7%)
Pneumonia with MTB	7/44 (16%)	14/87 (16%)	21/131 (16%)
MTB results not available[#]	2/44 (5%)	14/87 (16%)	16/131 (12%)
Pneumonia bacterial pathogens			
Streptococcus pneumoniae	0/35 (0%)	3/82 (4%)	3/117 (3%)
Staphylococcus aureus	0/35 (0%)	1/82 (1%)	1/117 (1%)
Contaminant	1/35 (3%)	4/82 (5%)	5/117 (4%)
Negative	34/35 (97%)	74/82 (90%)	108/117 (92%)
Pneumonia viral pathogens			
Inf A	0/39 (0%)	2/83 (2%)	2/122 (2%)
Inf B	1/39 (3%)	1/83 (1%)	2/122 (2%)
RSV	0/39 (0%)	1/83 (1%)	1/122 (1%)
Rhinovirus	0/39 (0%)	1/83 (1%)	1/122 (1%)
SARS-CoV-2	4/39 (10%)	0/83 (0%)	4/122 (3%)
Parainfluenza	1/39 (3%)	0/83 (0%)	1/122 (1%)
Negative	33/39 (85%)	78/83 (94%)	111/122 (91%)
**TB diagnosis**			
TB diagnosis (clinical/radiological/microbiological diagnosis)	31/140 (22%)	60/176 (34%)	91/316 (29%)
Known TB diagnosis	3/140 (2%)	7/176 (4%)	10/316 (3%)
New TB diagnosis	28/140 (20%)	53/176 (30%)	81/316 (26%)
Clinical TB diagnosis	16/140 (11%)	40/176 (23%)	56/316 (18%)
Radiological TB diagnosis	11/140 (8%)	17/176 (10%)	28/316 (9%)
Insufficient imaging available	17/140 (12%)	17/176 (10%)	34/316 (11%)
Microbiologically confirmed TB	20/140 (14%)	25/176 (14%)	45/316 (14%)
Xpert TB			
Positive	6/140 (4%)	14/176 (8%)	20/316 (6%)
Negative	82/140 (59%)	41/176 (23%)	123/316 (39%)
Participant unable to expectorate	52/140 (37%)	121/176 (69%)	173/316 (55%)
Urine LAM			
Positive	15/140 (11%)	11/176 (6%)	26/316 (8%)
Negative	24/140 (17%)	13/176 (7%)	37/316 (12%)
Sample not indicated	94/140 (67%)	102/176 (58%)	196/316 (62%)
Sample not taken PLHIV	7/140 (5%)	50/176 (28%)	57/316 (18%)
TB site			
Clinical/radiological pulmonary TB	22/140 (16%)	47/176 (27%)	69/316 (22%)
Pulmonary TB - microbiologically confirmed	6/140 (4%)	14/176 (8%)	20/316 (6%)
Disseminated TB	15/140 (11%)	11/176 (6%)	26/316 (8%)

*Diagnostic definition met according to *a priori* criteria. Subclassification not possible due to limited data as indicated in this table.
[#] Participants not able to expectorate among patients living with HIV and urine LAM result not available.

Figure S 3. Euler plot showing overlap between cases of pneumonia and TB

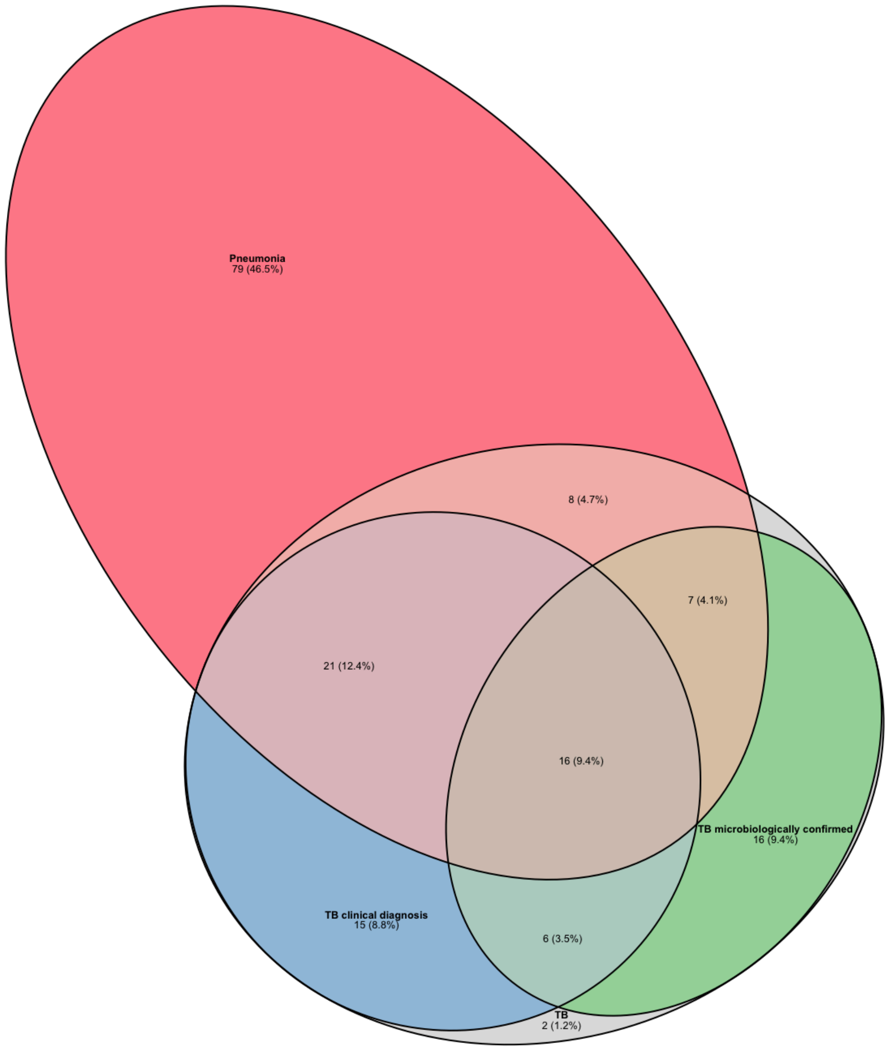

This plot demonstrates that we identified 39 patients with TB who did not have a diagnosis of pneumonia (participants did not have the requisite imaging findings and/or symptoms to be diagnosed with pneumonia per Infectious Diseases Society of America (IDSA)/American Thoracic Society (ATS) Consensus Guidelines[1]; see 'Pneumonia Diagnostic Criteria' above). This reflects 16 patients with a microbiological diagnosis, but no evidence of consolidation; 6 patients with positive microbiology and a clinical diagnosis of TB but no evidence of consolidation; 15 patients with a clinical recorded diagnosis and/or being treated for TB, but no evidence of consolidation; and 2 patients with radiological diagnosis of TB, but did not present with the symptoms required for our *a priori* definition of pneumonia (per IDSA/ATS criteria).

Note: the two participants with TB in the grey section of this plot were diagnosed radiologically (but did not have symptoms that aligned with the IDSA/ATS criteria definition of pneumonia). The 8 participants with TB and pneumonia (light pink section of the plot) were diagnosed with TB radiologically, and had symptoms present that aligned with the ERS/ATS definition of pneumonia.

Table S 20. Echocardiographic evidence of cardiac abnormalities

	Chiradzulu	QECH	Total
Number of echocardiograms performed	134/140 (96%)	170/176 (97%)	304/316 (96%)
Structural cardiac abnormality			
Present	84/134 (63%)	99/170 (58%)	183/304 (60%)
Absent	50/134 (37%)	71/170 (42%)	121/304 (40%)
Heart failure by ejection fraction			
HFrEF	33/134 (25%)	32/170 (19%)	65/304 (21%)
HFmrEF	8/134 (6%)	7/170 (4%)	15/304 (5%)
HFpEF	10/134 (7%)	20/170 (12%)	30/304 (10%)
Ischaemic cardiomyopathy			
Present	2/134 (1%)	1/170 (1%)	3/304 (1%)
Absent	132/134 (99%)	169/170 (99%)	301/304 (99%)
Hypertensive heart disease			
Present	25/134 (19%)	25/170 (15%)	50/304 (16%)
Absent	108/134 (81%)	140/170 (82%)	248/304 (82%)
Incomplete diagnostic information	1/134 (1%)	5/170 (3%)	6/304 (2%)
LV hypertrophy			
Present	26/134 (19%)	27/170 (16%)	53/304 (17%)
Absent	106/134 (79%)	133/170 (78%)	239/304 (79%)
Incomplete diagnostic information	2/134 (1%)	10/170 (6%)	12/304 (4%)
LV geometry			
Normal geometry	46/134 (34%)	60/170 (35%)	106/304 (35%)
Concentric remodelling	60/134 (45%)	73/170 (43%)	133/304 (44%)
Concentric hypertrophy	12/134 (9%)	15/170 (9%)	27/304 (9%)
Eccentric hypertrophy	14/134 (10%)	12/170 (7%)	26/304 (9%)
Incomplete diagnostic information	2/134 (1%)	10/170 (6%)	12/304 (4%)
Diastolic dysfunction			
LVDF with normal filling pressures	28/134 (21%)	27/170 (16%)	55/304 (18%)
LVDF with elevated filling pressures	29/134 (22%)	29/170 (17%)	58/304 (19%)
Normal LV diastolic function	68/134 (51%)	98/170 (58%)	166/304 (55%)
Incomplete diagnostic information	9/134 (7%)	16/170 (9%)	25/304 (8%)
Dilated cardiomyopathy			
Present	22/134 (16%)	28/170 (16%)	50/304 (16%)
Absent	112/134 (84%)	142/170 (84%)	254/304 (84%)
Restrictive cardiomyopathy			
Present	2/134 (1%)	0/170 (0%)	2/304 (1%)
Absent	132/134 (99%)	170/170 (100%)	302/304 (99%)
Hypertrophic cardiomyopathy			
Present	2/134 (1%)	0/170 (0%)	2/304 (1%)
Absent	132/134 (99%)	170/170 (100%)	302/304 (99%)
Constrictive pericarditis			
Present	1/134 (1%)	1/170 (1%)	2/304 (1%)
Absent	133/134 (99%)	169/170 (99%)	302/304 (99%)
Pericardial effusion			
Present	25/134 (19%)	21/170 (12%)	46/304 (15%)
Absent	109/134 (81%)	149/170 (88%)	258/304 (85%)
Pericardial effusion size			
Small	21/134 (16%)	18/170 (11%)	39/304 (13%)
Moderate	2/134 (1%)	1/170 (1%)	3/304 (1%)
Large	2/134 (1%)	2/170 (1%)	4/304 (1%)
Significant valve disease			
Present	47/134 (35%)	52/170 (31%)	99/304 (33%)
Absent	87/134 (65%)	118/170 (69%)	205/304 (67%)
Mitral valve			
Degenerative	17/134 (13%)	31/170 (18%)	48/304 (16%)
Functional	11/134 (8%)	22/170 (13%)	33/304 (11%)
Prolapse	0/134 (0%)	3/170 (2%)	3/304 (1%)
Rheumatic	10/134 (7%)	5/170 (3%)	15/304 (5%)
Normal	96/134 (72%)	109/170 (64%)	205/304 (67%)
Aortic valve			
Congenital(inc.bicuspid)	2/134 (1%)	1/170 (1%)	3/304 (1%)
Degenerative	22/134 (16%)	28/170 (16%)	50/304 (16%)

Functional	0/134 (0%)	1/170 (1%)	1/304 (0%)
Prolapse	0/134 (0%)	1/170 (1%)	1/304 (0%)
Rheumatic	7/134 (5%)	3/170 (2%)	10/304 (3%)
Normal	103/134 (77%)	136/170 (80%)	239/304 (79%)
Tricuspid valve			
Degenerative	13/134 (10%)	16/170 (9%)	29/304 (10%)
Functional	16/134 (12%)	23/170 (14%)	39/304 (13%)
Infective (endocarditis)	1/134 (1%)	0/170 (0%)	1/304 (0%)
Prolapse	0/134 (0%)	2/170 (1%)	2/304 (1%)
Rheumatic	8/134 (6%)	1/170 (1%)	9/304 (3%)
Normal	96/134 (72%)	128/170 (75%)	224/304 (74%)
Pulmonary valve			
Degenerative	3/134 (2%)	2/170 (1%)	5/304 (2%)
Functional	1/134 (1%)	3/170 (2%)	4/304 (1%)
Prolapse	1/134 (1%)	0/170 (0%)	1/304 (0%)
Rheumatic	2/134 (1%)	0/170 (0%)	2/304 (1%)
Normal	127/134 (95%)	165/170 (97%)	292/304 (96%)
Rheumatic valvular disease			
Present	10/134 (7%)	5/170 (3%)	15/304 (5%)
Absent	124/134 (93%)	165/170 (97%)	289/304 (95%)
Cardiac thrombus			
Present	2/134 (1%)	4/170 (2%)	6/304 (2%)
Absent	132/134 (99%)	166/170 (98%)	298/304 (98%)
Right heart failure			
Normal RV function	104/134 (78%)	138/170 (81%)	242/304 (80%)
RHF secondary to LHF	23/134 (17%)	22/170 (13%)	45/304 (15%)
Isolated RHF	7/134 (5%)	10/170 (6%)	17/304 (6%)

Echocardiography conducted on 304 participants.
HFrEF: heart failure with reduced ejection fraction; HFmrEF: heart failure with mildly reduced ejection fraction; HFpEF: heart failure with preserved ejection fraction; LV: left ventricle; RV: right ventricle; RHF: right heart failure; LHF: left heart failure; LVDF: left ventricular diastolic failure.

Table S 21. Pulmonary hypertension diagnoses by echocardiography

	Chiradzulu	QECH	Total
Pulmonary hypertension			
Low probability of PH	97/134 (72%)	130/170 (76%)	227/304 (75%)
Intermediate probability of PH	15/134 (11%)	22/170 (13%)	37/304 (12%)
High probability of PH	22/134 (16%)	18/170 (11%)	40/304 (13%)
Pulmonary hypertension			
Pre-capillary PH	8/134 (6%)	5/170 (3%)	13/304 (4%)
Post-capillary PH	14/134 (10%)	13/170 (8%)	27/304 (9%)

Echocardiography conducted on 304 participants

Table S 22. Cardiac arrhythmia

	Chiradzulu	QECH	Total
Atrial arrhythmia	10/135 (7%)	4/172 (2%)	14/316 (5%)
Atrial fibrillation	8/140 (6%)	4/176 (2%)	12/316 (4%)
Atrial flutter	2/140 (1%)	0/176 (0%)	2/316 (1%)
No evidence of atrial arrhythmia	125/140 (89%)	168/176 (95%)	293/316 (93%)
Incomplete diagnostic information	5/140 (4%)	4/176 (2%)	9/316 (3%)

Table S 23. Myocardial infarction

	Chiradzulu	QECH	Total
Myocardial infarction			
Positive	8/140 (6%)	10/176 (6%)	18/316 (6%)
Negative	132/140 (94%)	166/176 (94%)	298/316 (94%)
Acute myocardial infarction			
Positive	2/140 (1%)	6/176 (3%)	8/316 (3%)
Negative	138/140 (99%)	170/176 (97%)	308/316 (97%)
Prior myocardial infarction			
Positive	6/140 (4%)	6/176 (3%)	12/316 (4%)
Negative	129/140 (92%)	166/176 (94%)	295/316 (93%)
Incomplete diagnostic data	5/140 (4%)	4/176 (2%)	9/316 (3%)

Table S 24. Obstructive lung disease

	Chiradzulu	QECH	Total
**Chronic obstructive pulmonary disease**			
Positive	11/140 (8%)	10/176 (6%)	21/316 (7%)
Negative	129/140 (92%)	166/176 (94%)	295/316 (93%)
COPD severity			
Mild impairment	4/140 (3%)	2/176 (1%)	6/316 (2%)
Moderate impairment	3/140 (2%)	1/176 (1%)	4/316 (1%)
Severe impairment	2/140 (1%)	3/176 (2%)	5/316 (2%)
Incomplete diagnostic data	2/140 (1%)	4/176 (2%)	6/316 (2%)
Does not meet COPD criteria	129/140 (92%)	166/176 (94%)	295/316 (93%)
mMRC Dyspnoea Scale - COPD			
Grade 0	2/11 (18%)	0/10 (0%)	2/21 (10%)
Grade 1	3/11 (27%)	4/10 (40%)	7/21 (33%)
Grade 2	2/11 (18%)	2/10 (20%)	4/21 (19%)
Grade 3	1/11 (9%)	1/10 (10%)	2/21 (10%)
Grade 4	3/11 (27%)	3/10 (30%)	6/21 (29%)
Asthma			
Present	14/140 (10%)	4/176 (2%)	18/316 (6%)
Absent	126/140 (90%)	172/176 (98%)	298/316 (94%)
Asthma (GINA Criteria)			
Present	3/140 (2%)	3/176 (2%)	6/316 (2%)
Absent	128/140 (91%)	140/176 (80%)	268/316 (85%)
Incomplete diagnostic data	9/140 (6%)	33/176 (19%)	42/316 (13%)
Current wheezing illness (ISAAC/GAN Criteria)			
Present	11/140 (8%)	49/176 (28%)	60/316 (19%)
Absent	129/140 (92%)	127/176 (72%)	256/316 (81%)
Severe wheezing (ISAAC/GAN criteria)			
Present	7/140 (5%)	27/176 (15%)	34/316 (11%)
Absent	133/140 (95%)	149/176 (85%)	282/316 (89%)
Previous diagnosis of asthma			
Present	16/140 (11%)	14/176 (8%)	30/316 (9%)
Absent	124/140 (89%)	162/176 (92%)	286/316 (91%)

Table S 25. Haematological and vascular causes of breathlessness

	Chiradzulu	QECH	Total
**Anaemia severity**			
Mild	19/132 (14%)	25/171 (15%)	44/303 (15%)
Moderate	20/132 (15%)	29/171 (17%)	49/303 (16%)
Severe	9/132 (14%)	5/171 (8%)	14/303 (11%)
Transfusion threshold (Hb<70)	9/132 (7%)	9/171 (5%)	18/303 (6%)
**Pulmonary embolism (PE)**			
Present	0/140 (0%)	1/176 (1%)	1/316 (0.3%)
Absent	140/140 (100%)	175/176 (99%)	315/315 (99.7%)
**PE diagnostic method**			
CT diagnosis	0/0 (0%)	1/1 (100%)	1/1 (100%)
Echocardiographic criteria	0/134 (0%)	0/170 (0%)	0/304 (0%)
Clinical diagnosis	0/140 (0%)	1/176 (1%)	1/316 (0.3%)
Not clinically suspected	140/140 (100%)	175/176 (99%)	315/316 (99.7%)
**PE severity**			
Submassive PE	0/140 (0%)	1/176 (1%)	1/316 (0.3%)

CT: computed tomography

Table S 26. Pleural disease

	Chiradzulu	QECH	Total
**Pleural effusion**	56/140 (40%)	62/176 (35%)	118/316 (37%)
Pleural effusion characteristics			
Simple	51/136 (38%)	56/174 (32%)	107/310 (35%)
Complex	5/136 (4%)	6/174 (3%)	11/310 (4%)
Pleural effusion size/depth			
< 2cm US / <2 ICS CXR*	103/136 (76%)	141/174 (81%)	244/310 (79%)
≥2cm US / ≥2 ICS CXR*	33/136 (24%)	33/174 (19%)	66/310 (21%)
**Pneumothorax**	1/140 (1%)	2/176 (1%)	3/316 (1%)
Tension pneumothorax			
Present	0/136 (0%)	1/174 (1%)	1/310 (0%)
Absent	1/136 (1%)	1/174 (1%)	2/310 (1%)

US: ultrasound; ICS: intercostal spaces; CXR: chest x-ray.
*2cm effusion depth on ultrasound or 2 intercostal spaces on chest x-ray.

Figure S 4. Euler plot showing overlap between pleural effusion with heart failure, pneumonia and TB.

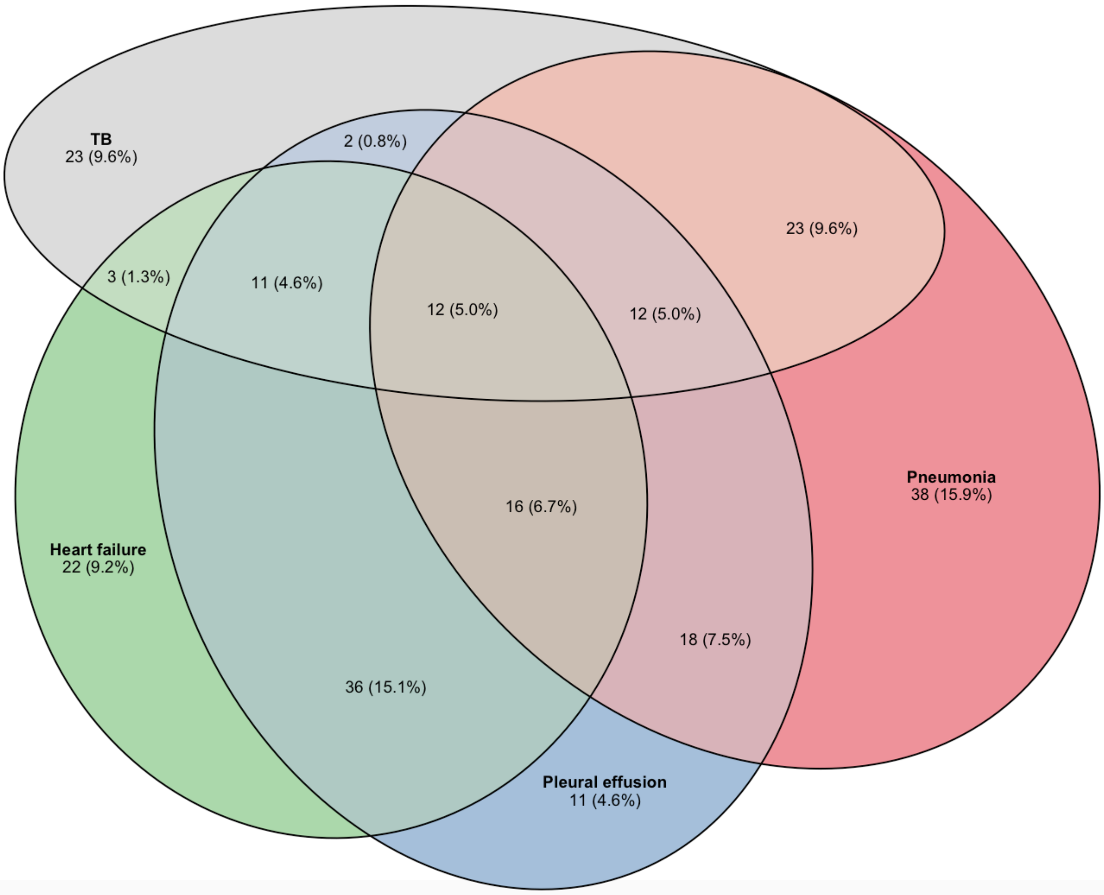

Section 5. Outcomes: whole cohort (Multilink)

Table S 27. Breathlessness and other risk factors for mortality among the entire medical cohort.

Variable	HR_{cr}	95% CI_{cr}	p-$value_{cr}$	B-H adjusted p-value	HR_{adj}	95% CI_{adj}	p-$value_{adj}$
Breathlessness	2.54	1.98-3.27	<0.0001	<0.0001	1.79	1.38-2.33	<0.0001
Age	1.03	1.02-1.04	<0.0001	<0.0001	1.02	1.02-1.03	<0.0001
Sex							
Female	Ref	Ref	Ref	Ref	Ref	Ref	Ref
Male	1.25	0.97-1.62	0.08	0.09	1.18	0.91-1.52	0.21
Universal vital assessment	1.22	1.16-1.29	<0.0001	<0.0001	1.17	1.11-1.24	<0.0001
Unwell period, weeks	1.05	1.03-1.07	<0.0001	<0.0001	1.04	1.01-1.06	0.002
HIV infection[#]	1.02	0.78-1.32	0.90	0.90	-	-	-

Results from univariable and multivariable Cox regression model. Multivariable model included adjustment for all variables shown in this Table, but not for HIV infection.
[#]HIV infection is a component of the UVA score, and we therefore did not adjust for HIV separately in the multivariable model due to collinearity.
B-H adjusted: Benjamini-Hochberg adjustment for multiple comparisons.

Section 6. Supplementary statistical methods: model development

Survival models were initially conducted with Cox models and tested for proportional hazards. If proportional hazards assumptions were found to be not met, we proceeded with flexible parametric survival models (Royston-Parmar models; details below) instead.

We *a priori* adjusted by age, sex and UVA in all multivariable models, based on clinical plausibility and following consensus agreement among authors.

In our model to assess the association between breathlessness and mortality among all medical admissions, we additionally adjusted for breathlessness and duration between onset of symptoms and hospital presentation (Table S25).

In order to understand the association between functional status and mortality, we adjusted by age, sex, UVA, in addition to HRQoL and conditions with greater than 10 events (deaths) per variable (heart failure, pneumonia, anaemia and tuberculosis; Table S30).

To understand the association between each condition and mortality, we adjusted by age, sex, UVA and heart failure, pneumonia, anaemia and tuberculosis (Tables S31-35).

Flexible parametric model: statistical details

In instances where Cox regression was not possible due to violation of the proportional hazards assumption, we used a flexible parametric model, using Stata command *stpm2*. The number of knots (degrees of freedom for the restricted cubic spline function), and the degrees of freedom for the variable with time varying effect (in our models, the variable with time varying effects was heart failure diagnosis), were chosen based on the model with the lowest Bayesian Information Criterion (BIC) and Akaike Information Criterion (AIC). We prioritised a low BIC to minimise to avoid an over-fitted model. We then plotted the predicted model against the crude Kaplan Meier plot to confirm a good model fit (Figure S2).

Survival predictions based on the flexible parametric model

Flexible parametric models were also used to predict survival, adjusting for the presence of co-existing pathologies to assess the impact of concurrent pathologies. These models included conditions with greater than 10 events (deaths) per variable: heart failure, pneumonia, anaemia and tuberculosis. We excluded pleural effusion and pulmonary hypertension in these models as they were most commonly due to heart or lung pathologies in this selected cohort of breathless patients.

We obtained survival estimates using the following approaches (Figure E3; 26Figure S3; Table S26):

1. Direct observed survival for each condition (no model).
2. Predicted mortality for each condition, setting covariate diagnoses (heart failure, anaemia, pneumonia and TB) to zero, except for the index condition. This flexible parametric model included the following covariates: heart failure, anaemia, pneumonia and TB.
3. Age and sex- standardised mortality for each condition (age and sex set to the mean of the cohort population). This flexible parametric model included the index condition as well as age and sex.
4. Age and sex- standardised mortality for each condition (age and sex set to the mean of the cohort population). In addition, covariate diagnoses (heart failure, anaemia, pneumonia and TB) to zero, except for the index condition. This flexible parametric model included covariates: heart failure, anaemia, pneumonia, TB, age and sex

Figure S 5. Flexible parametric model fit

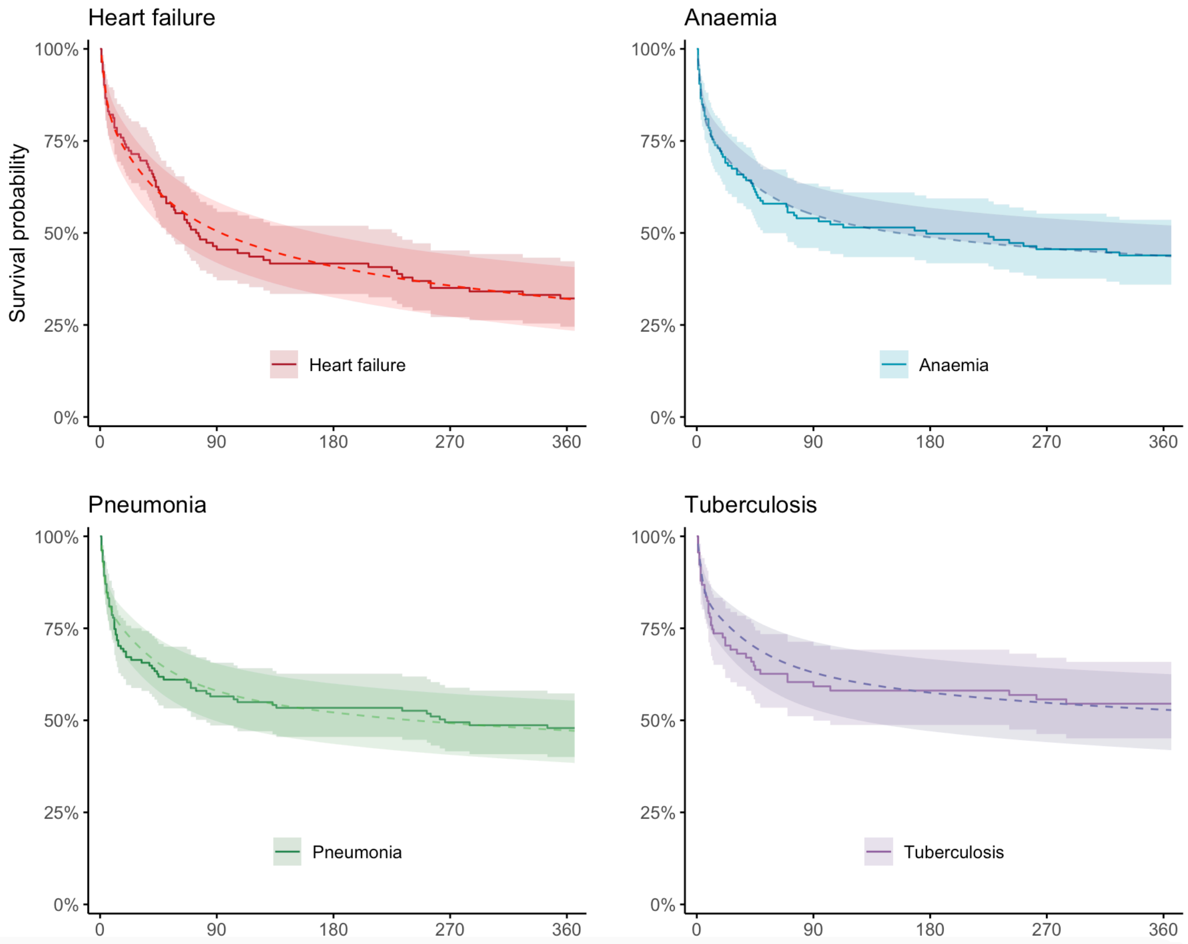

Solid line reflects direct survival estimates for participants with each condition (Kaplan-Meier plot).
Dashed line reflects predicted unadjusted survival from the flexible parametric model.

Section 7. Outcomes: breathless cohort

Table S 28. Unadjusted and adjusted 30-day, 90-day and one-year mortality for heart failure, anaemia, pneumonia and TB: results from flexible parametric model

Condition	30-day mortality (95% CI)	90-day mortality (95% CI)	1 year mortality (95% CI)
**Heart failure**			
Heart failure (cr) [a)]	29.46 (21.69 - 38.64)	54.46 (45.09 - 63.53)	68.80 (59.41 - 76.86)
Heart failure (single disease) [b)]	29.20 (21.75 - 38.51)	44.83 (35.55 - 55.31)	63.25 (52.23 - 74.25)
Heart failure (age and sex adjusted) [c)]	29.54 (22.16 - 36.22)	44.96 (36.55 - 52.26)	62.97 (53.13 - 70.75)
Heart failure (single disease, age and sex adjusted) [d)]	24.66 (16.90 - 31.69)	38.58 (28.68 - 47.1)	55.86 (43.34 - 65.61)
**Anaemia**			
Anaemia (cr) [a)]	32.53 (24.87 - 41.27)	46.03 (37.45 - 54.85)	57.38 (48.37 - 65.91)
Anaemia (single disease) [b)]	28.98 (21.19 - 38.85)	37.62 (28.50 - 48.52)	45.03 (34.82 - 56.69)
Anaemia (age and sex adjusted) [c)]	31.91 (24.90 - 38.27)	44.20 (36.04 - 51.32)	55.38 (46.48 - 62.80)
Anaemia (single disease, age and sex adjusted) [d)]	28.51 (19.48 - 36.53)	36.86 (26.50 - 45.76)	44.13 (32.58 - 53.70)
**Pneumonia**			
Pneumonia (cr) [a)]	33.59 (25.97 - 42.17)	43.51 (35.21 - 52.19)	52.71 (44.02 - 61.24)
Pneumonia (single disease) [b)]	27.49 (20.08 - 36.94)	35.82 (27.12 - 46.29)	43.00 (33.26 - 54.23)
Pneumonia (age and sex adjusted) [c)]	31.81 (24.65 - 38.29)	44.00 (35.73 - 51.21)	54.99 (46.15 - 62.37)
Pneumonia (single disease, age and sex adjusted) [d)]	29.87 (20.73 - 37.96)	38.48 (28.18 - 47.30)	45.92 (34.68 - 55.22)
**TB**			
TB (cr) [a)]	30.77 (22.06 - 41.10)	40.66 (30.96 - 51.15)	47.12 (36.77 - 57.73)
TB (single disease) [b)]	17.55 (11.34 - 26.61)	23.36 (15.53 - 34.26)	28.64 (19.38 - 41.05)
TB (age and sex adjusted adj) [c)]	28.36 (20.21 - 35.68)	39.72 (29.74 - 48.28)	50.36 (39.20 - 59.46)
TB (single disease, age and sex adjusted) [d)]	19.77 (11.07 - 27.61)	26.13 (15.42 - 35.48)	31.94 (19.46 - 42.49)

[a)] These are direct survival estimates for participants with each condition.
[b)] Estimates obtained from flexible parametric model (FPM). This model included covariates: heart failure, anaemia, pneumonia and TB. For the prediction, all covariates were set to zero, except for the index condition.
[c)] Estimates obtained from FPM. This model included the index condition as well as age and sex. For the prediction, age and sex were set to the mean in the cohort population.
[d)] Estimates obtained from FPM. This model included covariates: heart failure, anaemia, pneumonia and TB, as well as age and sex. For the prediction, age and sex were set to the mean in the cohort population and all conditions were set to zero, except for the index condition.

Figure S 6. Kaplan Meier survival plots for all aetiologies

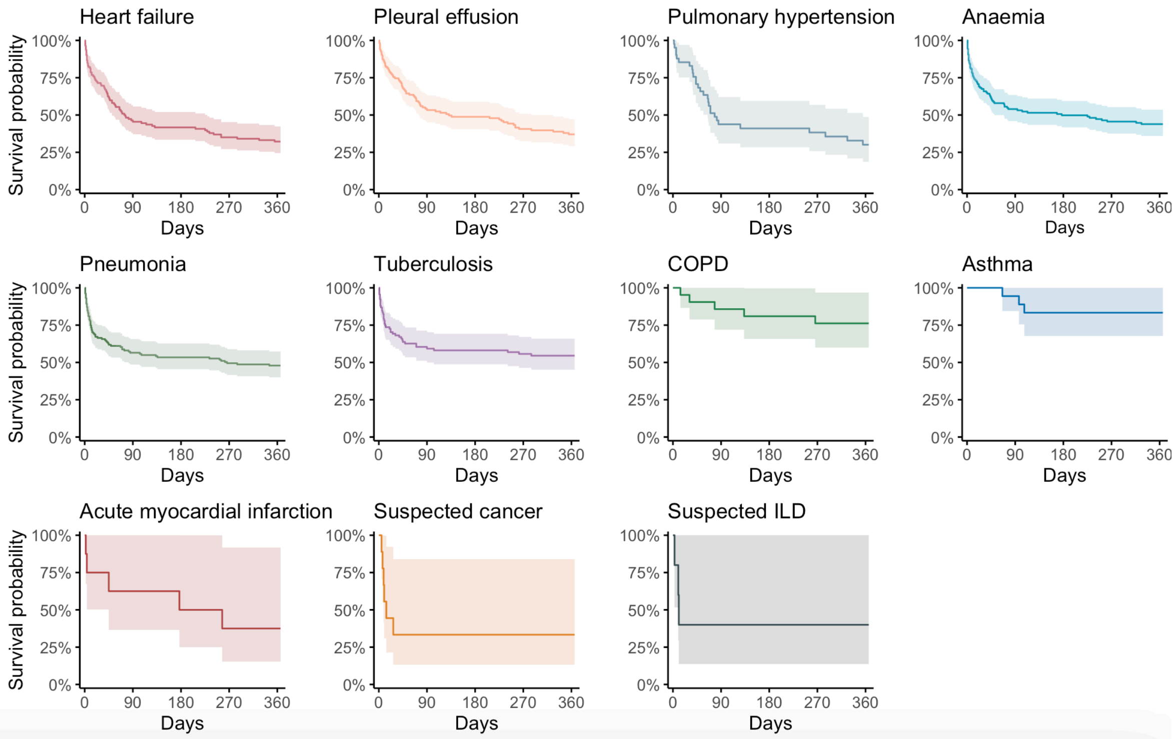

Figure S 7. Survival plot sensitivity analysis

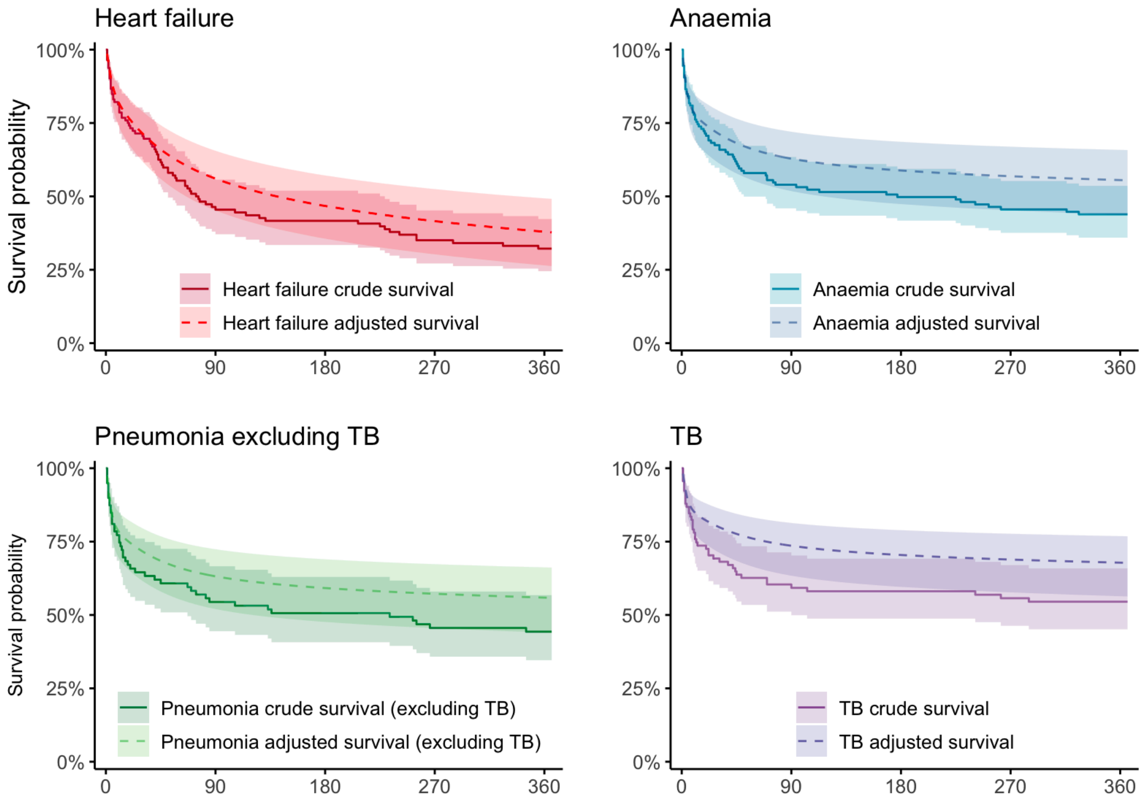

In this sensitivity we excluded cases of TB-associated pneumonia from the pneumonia plot (52 out of 131 cases of pneumonia were TB-associated; see Figure S3 for further details). This sensitivity analysis demonstrates consistent findings to the plot presented in Figure 2 in the manuscript.

Table S 29. In-hospital, 30-day, 90-day and 1-year mortality, stratified by demographic factors, HIV infection status, functional measures, and diagnoses

Variable	Variable category	In-hospital mortality	30-day mortality	90-day mortality	1-year mortality
Demographic and patient factors					
Age	18-29	5/37 (13.5%)	5/37 (13.5%)	7/37 (18.9%)	7/36 (19.4%)
	30-39	14/61 (23%)	16/61 (26.2%)	22/61 (36.1%)	24/60 (40%)
	40-49	13/52 (25%)	15/52 (28.8%)	21/52 (40.4%)	25/49 (51%)
	50-59	10/46 (21.7%)	13/46 (28.3%)	19/46 (41.3%)	22/45 (48.9%)
	60-69	10/52 (19.2%)	14/52 (26.9%)	21/52 (40.4%)	32/49 (65.3%)
	70-79	14/50 (28%)	18/50 (36%)	29/50 (58%)	35/50 (70%)
	80-89	5/14 (35.7%)	6/14 (42.9%)	7/14 (50%)	10/14 (71.4%)
	≥90	1/4 (25%)	1/4 (25%)	2/4 (50%)	2/4 (50%)
Sex	Female	22/113 (19.5%)	28/113 (24.8%)	37/113 (32.7%)	50/111 (45%)
	Male	50/203 (24.6%)	60/203 (29.6%)	91/203 (44.8%)	107/196 (54.6%)
UVA	Low risk (UVA 0-1)	12/99 (12.1%)	18/99 (18.2%)	34/99 (34.3%)	44/93 (47.3%)
	Medium risk (UVA 2-4)	33/170 (19.4%)	43/170 (25.3%)	62/170 (36.5%)	78/167 (46.7%)
	High risk (UVA >4)	27/47 (57.4%)	27/47 (57.4%)	32/47 (68.1%)	35/47 (74.5%)
Functional outcomes					
MRC	Grade 0	20/75 (26.7%)	24/75 (32%)	29/75 (38.7%)	35/70 (50%)
	Grade 1	8/69 (11.6%)	11/69 (15.9%)	23/69 (33.3%)	28/69 (40.6%)
	Grade 2	9/67 (13.4%)	14/67 (20.9%)	21/67 (31.3%)	26/64 (40.6%)
	Grade 3	4/21 (19%)	5/21 (23.8%)	11/21 (52.4%)	13/21 (61.9%)
	Grade 4	31/84 (36.9%)	34/84 (40.5%)	44/84 (52.4%)	55/83 (66.3%)
CFS	Not frail (CFS ≤4)0	10/124 (8.1%)	15/124 (12.1%)	34/124 (27.4%)	46/121 (38%)
	Frail (CFS 5-6)5	36/137 (26.3%)	44/137 (32.1%)	56/137 (40.9%)	68/131 (51.9%)
	Severely frail (CFS ≥7)	26/55 (47.3%)	29/55 (52.7%)	38/55 (69.1%)	43/55 (78.2%)
Disability	With Disability	52/170 (30.6%)	59/170 (34.7%)	89/170 (52.4%)	107/167 (64.1%)
	Without Disability	20/146 (13.7%)	29/146 (19.9%)	39/146 (26.7%)	50/140 (35.7%)
HIV infection					
HIV infection	HIV uninfected	39/196 (19.9%)	48/196 (24.5%)	78/196 (39.8%)	100/189 (52.9%)
	HIV infected	29/116 (25%)	36/116 (31%)	46/116 (39.7%)	53/114 (46.5%)
	Status unknown	4/4 (100%)	4/4 (100%)	4/4 (100%)	4/4 (100%)
Causes of breathlessness					
Pneumonia	Absent	34/185 (18.4%)	44/185 (23.8%)	71/185 (38.4%)	89/178 (50%)
	Present	38/131 (29%)	44/131 (33.6%)	57/131 (43.5%)	68/129 (52.7%)
TB	Absent	48/225 (21.3%)	60/225 (26.7%)	91/225 (40.4%)	116/220 (52.7%)
	Present	24/91 (26.4%)	28/91 (30.8%)	37/91 (40.7%)	41/87 (47.1%)
Heart failure	Absent	47/204 (23%)	55/204 (27%)	67/204 (32.8%)	82/198 (41.4%)
	Present	25/112 (22.3%)	33/112 (29.5%)	61/112 (54.5%)	75/109 (68.8%)
Anaemia	Absent	38/190 (20%)	47/190 (24.7%)	70/190 (36.8%)	87/185 (47%)
	Present	34/126 (27%)	41/126 (32.5%)	58/126 (46%)	70/122 (57.4%)
Acute myocardial infarction	Absent	70/308 (22.7%)	86/308 (27.9%)	125/308 (40.6%)	152/299 (50.8%)
	Present	2/8 (25%)	2/8 (25%)	3/8 (37.5%)	5/8 (62.5%)
Pulmonary hypertension	Absent	68/275 (24.7%)	82/275 (29.8%)	105/275 (38.2%)	129/268 (48.1%)
	Present	4/41 (9.8%)	6/41 (14.6%)	23/41 (56.1%)	28/39 (71.8%)
COPD	Absent	71/295 (24.1%)	87/295 (29.5%)	125/295 (42.4%)	152/286 (53.1%)
	Present	1/21 (4.8%)	1/21 (4.8%)	3/21 (14.3%)	5/21 (23.8%)
Asthma	Absent	72/298 (24.2%)	88/298 (29.5%)	127/298 (42.6%)	154/290 (53.1%)
	Present	0/18 (0%)	0/18 (0%)	1/18 (5.6%)	3/17 (17.6%)
Pleural effusion	Absent	49/198 (24.7%)	58/198 (29.3%)	73/198 (36.9%)	84/193 (43.5%)
	Present	23/118 (19.5%)	30/118 (25.4%)	55/118 (46.6%)	73/114 (64%)
Pneumothorax	Absent	72/313 (23%)	88/313 (28.1%)	128/313 (40.9%)	156/305 (51.1%)
	Present	0/3 (0%)	0/3 (0%)	0/3 (0%)	1/2 (50%)
Pulmonary embolism	Absent	72/315 (22.9%)	88/315 (27.9%)	128/315 (40.6%)	157/306 (51.3%)
	Present	0/1 (0%)	0/1 (0%)	0/1 0%)	0/1 (0%)

Table S 30. HIV status and one-year mortality by diagnosis.

Variable	HIV negative	PLHIV	One-year mortality HIV negative	One-year mortality PLHIV
Pneumonia	68/131 (51.9%)	62/131 (47.3%)	38/68 (55.9%)	29/62 (46.8%)
TB	34/91 (37.4%)	56/91 (61.5%)	11/34 (32.4%)	29/56 (51.8%)
Heart failure	82/112 (73.2%)	28/112 (25%)	53/82 (64.6%)	20/28 (71.4%)
Anaemia	75/126 (59.5%)	50/126 (39.7%)	43/75 (57.3%)	26/50 (52%)
Myocardial infarction	5/8 (62.5%)	3/8 (37.5%)	4/5 (80%)	1/3 (33.3%)
Pulmonary hypertension	29/41 (70.7%)	12/41 (29.3%)	20/29 (69%)	8/12 (66.7%)
COPD	16/21 (76.2%)	5/21 (23.8%)	3/16 (18.8%)	2/5 (40%)
Asthma	11/18 (61.1%)	7/18 (38.9%)	1/11 (9.1%)	2/7 (28.6%)
Pleural effusion	83/118 (70.3%)	34/118 (28.8%)	51/83 (61.4%)	21/34 (61.8%)
Pneumothorax	3/3 (100%)	0/3 (0%)	1/3 (33.3%)	0/0 (0%)
Pulmonary embolism	0/1 (0%)	1/1 (100%)	0/0 (0%)	0/1 (0%)

Note: We were unable to ascertain HIV status for four participants within our cohort

Table S 31. Association between functional and demographic factors and mortality. Univariable flexible parametric models

Variable	HR (95% CI)	p-value	B-H adjusted p-value
mMRC	1.16 (1.04-1.29)	0.01	0.03
UVA	1.15 (1.07-1.24)	<0.0001	<0.0001
CFS	1.33 (1.20-1.47)	<0.0001	<0.0001
Disability	2.27 (1.62-3.18)	<0.0001	<0.0001
Duration from symptom onset to admission, weeks	1.04 (1.01-1.07)	0.01	0.02
HIV infection	0.91 (0.65-1.26)	0.56	0.56
Age*	1.02 (1.01-1.03)	<0.0001	<0.0001
Sex	1.30 (0.93-1.82)	0.12	0.14

Results are from univariable flexible parametric model. B-H adjusted: Benjamini-Hochberg adjustment for multiple comparisons.

Table S 32. Association between UVA, age, sex, heart failure, anaemia, pneumonia, TB and mortality: Flexible parametric model

Variable		HR (95% CI)	Unadjusted p-value	Adjusted HR (95% CI)	Adjusted p-value
UVA*		1.15 (1.07-1.24)	0.0001	1.17 (1.09-1.26)	<0.0001
Age*		1.02 (1.01-1.03)	<0.0001	1.02 (1.01-1.03)	<0.0001
Sex	Female	Ref	Ref	Ref	Ref
	Male	1.30 (0.93-1.82)	0.12	1.23 (0.87-1.73)	0.24
Heart failure	Absent	Ref	Ref	Ref	Ref
	Present	1.61 (1.16-2.24)	0.004	1.63 (1.16-2.30)	0.005
Anaemia	Absent	Ref	Ref	Ref	Ref
	Present	1.33 (0.97-1.82)	0.08	1.30 (0.94-1.79)	0.12
Pneumonia	Absent	Ref	Ref	Ref	Ref
	Present	1.16 (0.85-1.59)	0.36	1.33 (0.95-1.86)	0.10
TB	Absent	Ref	Ref	Ref	Ref
	Present	0.85 (0.60-1.22)	0.39	0.79 (0.54-1.15)	0.21

Multivariable model with adjustment for all variables shown in this table.
*Age and UVA included as continuous variables

Table S 33. EQ5D-5L results by category at each follow-up

	Hospital admission	Day 30	Day 90	1 Year
EQ5D Mobility				
No problems	20/316 (6%)	124/195 (64%)	117/168 (70%)	87/150 (58%)
Slight problems	94/316 (30%)	30/195 (15%)	23/168 (14%)	50/150 (33%)
Moderate problems	104/316 (33%)	22/195 (11%)	15/168 (9%)	8/150 (5%)
Severe problems	51/316 (16%)	15/195 (8%)	7/168 (4%)	5/150 (3%)
Unable to do	47/316 (15%)	4/195 (2%)	6/168 (4%)	0/150 (0%)
EQ5D Self care				
No problems	33/316 (10%)	129/195 (66%)	135/168 (80%)	104/150 (69%)
Slight problems	129/316 (41%)	35/195 (18%)	16/168 (10%)	35/150 (23%)
Moderate problems	76/316 (24%)	19/195 (10%)	10/168 (6%)	7/150 (5%)
Severe problems	41/316 (13%)	10/195 (5%)	3/168 (2%)	4/150 (3%)
Unable to do	37/316 (12%)	2/195 (1%)	4/168 (2%)	0/150 (0%)
EQ5D Usual activities				
No problems	15/316 (5%)	101/195 (52%)	117/168 (70%)	76/150 (51%)
Slight problems	67/316 (21%)	45/195 (23%)	28/168 (17%)	56/150 (37%)
Moderate problems	131/316 (41%)	31/195 (16%)	14/168 (8%)	14/150 (9%)
Severe problems	59/316 (19%)	16/195 (8%)	2/168 (1%)	4/150 (3%)
Unable to do	44/316 (14%)	2/195 (1%)	7/168 (4%)	0/150 (0%)
EQ5D Pain/Discomfort				
No symptoms	73/316 (23%)	132/195 (68%)	111/168 (66%)	109/150 (73%)
Slight symptoms	92/316 (29%)	36/195 (18%)	27/168 (16%)	25/150 (17%)
Moderate symptoms	102/316 (32%)	22/195 (11%)	28/168 (17%)	16/150 (11%)
Severe symptoms	42/316 (13%)	4/195 (2%)	1/168 (1%)	0/150 (0%)
Extreme symptoms	7/316 (2%)	1/195 (1%)	1/168 (1%)	0/150 (0%)
EQ5D Anxiety/Depression				
No symptoms	116/316 (37%)	142/195 (73%)	139/168 (83%)	115/150 (77%)
Slight symptoms	118/316 (37%)	34/195 (17%)	20/168 (12%)	27/150 (18%)
Moderate symptoms	57/316 (18%)	16/195 (8%)	4/168 (2%)	6/150 (4%)
Severe symptoms	24/316 (8%)	3/195 (2%)	5/168 (3%)	1/150 (1%)
Extreme symptoms	1/316 (0%)	0/195 (0%)	0/168 (0%)	1/150 (1%)

Figure S 8. Distribution of HRQoL health utility scores among survivors at each follow-up

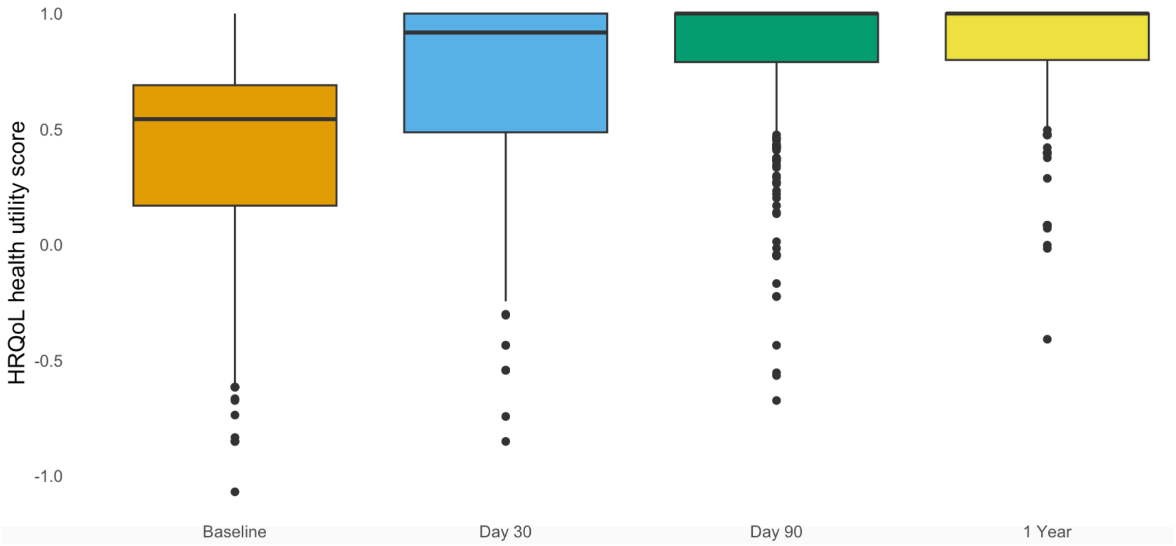

Table S 34. Demographic details, functional and clinical outcomes among participants with heart failure

Heart failure	Hospital	Day 30	Day 90	1 Year
**Mean age, years [SD]**	58 [18]			
**Sex**				
Female	33/112 (29%)			
Male	79/112 (71%)			
**Mortality**	26/112 (23%)	33/112 (29%)	61/112 (54%)	75/109 (69%)
**Readmission** (survivors) [§]	NA	NA	9/47 (19%)	6/34 (18%)
**Hospital length of stay (LOS)**				
LOS (all participants), median days [IQR]	6 [3.75-9.25]	NA	NA	NA
LOS (survivors), median days [IQR]	6.5 [4-9]	NA	NA	NA
**NYHA**				
NYHA, median [IQR]	3 [2-4]	NA	NA	1 [1-2]
NYHA Class 1	19/112 (17%)	NA	NA	18/34 (53%)
NYHA Class 2	17/112 (15%)	NA	NA	13/34 (38%)
NYHA Class 3	40/112 (36%)	NA	NA	3/34 (9%)
NYHA Class 4	36/112 (32%)	NA	NA	0/34 (0%)
**mMRC**				
mMRC, median [IQR]	2 [1-4]	NA	NA	0 [0-1]
mMRC Grade 0	16/112 (14%)	NA	NA	20/34 (59%)
mMRC Grade 1	24/112 (21%)	NA	NA	7/34 (21%)
mMRC Grade 2	26/112 (23%)	NA	NA	7/34 (21%)
mMRC Grade 3	9/112 (8%)	NA	NA	0/34 (0%)
mMRC Grade 4	37/112 (33%)	NA	NA	0/34 (0%)
**Disability**				
With Disability	69/112 (62%)	NA	5/44 (11%)	NA
Without disability	43/112 (38%)	NA	39/44 (89%)	NA
**EQ5D-5L**				
HRQoL health utility score, median [IQR]	0.43 [0.13-0.65]	0.76 [0.54-1]	0.86 [0.63-1]	0.88 [0.76-1]
EQ5D-VAS, median [SD]	51.18 (15.8)	70.3 (19.11)	75.16 (18.16)	76.56 (17.73)
EQ5D Mobility				
No problems	2/112 (2%)	29/60 (48%)	23/44 (52%)	20/34 (59%)
Slight problems	26/112 (23%)	13/60 (22%)	9/44 (20%)	11/34 (32%)
Moderate problems	41/112 (37%)	8/60 (13%)	6/44 (14%)	1/34 (3%)
Severe problems	23/112 (21%)	8/60 (13%)	3/44 (7%)	2/34 (6%)
Unable to do	20/112 (18%)	2/60 (3%)	3/44 (7%)	0/34 (0%)
EQ5D Self care				
No problems	9/112 (8%)	30/60 (50%)	30/44 (68%)	24/34 (71%)
Slight problems	38/112 (34%)	15/60 (25%)	7/44 (16%)	9/34 (26%)
Moderate problems	31/112 (28%)	8/60 (13%)	4/44 (9%)	0/34 (0%)
Severe problems	19/112 (17%)	6/60 (10%)	1/44 (2%)	1/34 (3%)
Unable to do	15/112 (13%)	1/60 (2%)	2/44 (5%)	0/34 (0%)
EQ5D Usual activities				
No problems	2/112 (2%)	21/60 (35%)	23/44 (52%)	14/34 (41%)
Slight problems	20/112 (18%)	14/60 (23%)	12/44 (27%)	17/34 (50%)
Moderate problems	41/112 (37%)	15/60 (25%)	6/44 (14%)	2/34 (6%)
Severe problems	30/112 (27%)	8/60 (13%)	1/44 (2%)	1/34 (3%)
Unable to do	19/112 (17%)	2/60 (3%)	2/44 (5%)	0/34 (0%)
EQ5D Pain/Discomfort				
No symptoms	30/112 (27%)	34/60 (57%)	22/44 (50%)	23/34 (68%)
Slight symptoms	31/112 (28%)	15/60 (25%)	13/44 (30%)	9/34 (26%)
Moderate symptoms	35/112 (31%)	9/60 (15%)	9/44 (20%)	2/34 (6%)
Severe symptoms	14/112 (12%)	1/60 (2%)	0/44 (0%)	0/34 (0%)
Extreme symptoms	2/112 (2%)	1/60 (2%)	0/44 (0%)	0/34 (0%)
EQ5D Anxiety/Depression				
No symptoms	37/112 (33%)	35/60 (58%)	32/44 (73%)	23/34 (68%)
Slight symptoms	42/112 (38%)	17/60 (28%)	11/44 (25%)	8/34 (24%)
Moderate symptoms	24/112 (21%)	7/60 (12%)	0/44 (0%)	3/34 (9%)
Severe symptoms	9/112 (8%)	1/60 (2%)	1/44 (2%)	0/34 (0%)
Extreme symptoms	0/112 (0%)	0/60 (0%)	0/44 (0%)	0/34 (0%)

[§]Number of participants who survived to follow-up and had at least one hospital admission between the index admission and follow-up.

Table S 35. Demographic details, functional and clinical outcomes among participants with anaemia

Anaemia	Hospital	Day 30	Day 90	1 Year
**Mean age, years [SD]**	51 [18]			
**Sex**				
Female	41/126 (33%)			
Male	85/126 (67%)			
**Mortality**	35/125 (28%)	41/126 (33%)	58/126 (46%)	70/122 (57%)
**Readmission** (survivors) [§]	NA	NA	5/65 (8%)	10/51 (20%)
**Hospital length of stay (LOS)**				
LOS (all participants), median days [IQR]				
LOS (survivors), median days [IQR]	6.5 [3-10]	NA	NA	NA
**mMRC**				
mMRC, median [IQR]	2 [1-4]	NA	NA	0 [0-1]
mMRC Grade 0	23/126 (18%)	NA	NA	35/52 (67%)
mMRC Grade 1	29/126 (23%)	NA	NA	12/52 (23%)
mMRC Grade 2	34/126 (27%)	NA	NA	5/52 (10%)
mMRC Grade 3	7/126 (6%)	NA	NA	0/52 (0%)
mMRC Grade 4	33/126 (26%)	NA	NA	0/52 (0%)
**Disability**				
With Disability	72/126 (57%)	NA	6/64 (9%)	NA
Without disability	54/126 (43%)	NA	58/64 (91%)	NA
**EQ5D-5L**				
HRQoL health utility score, median [IQR]	0.53 [0.23-0.66]	1 [0.72-1]	0.94 [0.71-1]	0.95 [0.76-1]
EQ5D-VAS, median [SD]	51.29 (14.42)	75.45 (18.72)	77.38 (18.6)	78.87 (17.06)
EQ5D Mobility				
No problems	4/126 (3%)	43/67 (64%)	43/64 (67%)	32/52 (62%)
Slight problems	38/126 (30%)	10/67 (15%)	7/64 (11%)	16/52 (31%)
Moderate problems	48/126 (38%)	7/67 (10%)	8/64 (12%)	3/52 (6%)
Severe problems	16/126 (13%)	6/67 (9%)	4/64 (6%)	1/52 (2%)
Unable to do	20/126 (16%)	1/67 (1%)	2/64 (3%)	0/52 (0%)
EQ5D Self care				
No problems	10/126 (8%)	44/67 (66%)	51/64 (80%)	36/52 (69%)
Slight problems	54/126 (43%)	11/67 (16%)	5/64 (8%)	13/52 (25%)
Moderate problems	33/126 (26%)	9/67 (13%)	5/64 (8%)	2/52 (4%)
Severe problems	16/126 (13%)	3/67 (4%)	2/64 (3%)	1/52 (2%)
Unable to do	13/126 (10%)	0/67 (0%)	1/64 (2%)	0/52 (0%)
EQ5D Usual activities				
No problems	3/126 (2%)	36/67 (54%)	40/64 (62%)	28/52 (54%)
Slight problems	26/126 (21%)	16/67 (24%)	14/64 (22%)	18/52 (35%)
Moderate problems	60/126 (48%)	10/67 (15%)	6/64 (9%)	5/52 (10%)
Severe problems	22/126 (17%)	5/67 (7%)	1/64 (2%)	1/52 (2%)
Unable to do	15/126 (12%)	0/67 (0%)	3/64 (5%)	0/52 (0%)
EQ5D Pain/Discomfort				
No symptoms	34/126 (27%)	48/67 (72%)	40/64 (62%)	36/52 (69%)
Slight symptoms	39/126 (31%)	8/67 (12%)	10/64 (16%)	9/52 (17%)
Moderate symptoms	33/126 (26%)	10/67 (15%)	12/64 (19%)	7/52 (13%)
Severe symptoms	17/126 (13%)	1/67 (1%)	1/64 (2%)	0/52 (0%)
Extreme symptoms	3/126 (2%)	0/67 (0%)	1/64 (2%)	0/52 (0%)
EQ5D Anxiety/Depression				
No symptoms	43/126 (34%)	51/67 (76%)	54/64 (84%)	37/52 (71%)
Slight symptoms	45/126 (36%)	11/67 (16%)	9/64 (14%)	12/52 (23%)
Moderate symptoms	29/126 (23%)	5/67 (7%)	1/64 (2%)	3/52 (6%)
Severe symptoms	8/126 (6%)	0/67 (0%)	0/64 (0%)	0/52 (0%)
Extreme symptoms	1/126 (1%)	0/67 (0%)	0/64 (0%)	0/52 (0%)

[§]Number of participants who survived to follow-up and had at least one hospital admission between the index admission and follow-up.

Table S 36. Demographic details, functional and clinical outcomes among participants with pneumonia

Pneumonia	Hospital Discharge	Day 30	Day 90	1 Year
**Mean age, years [SD]**	48 [16.5]	NA	NA	NA
**Sex**				
Female	37/131 (28%)	NA	NA	NA
Male	94/131 (72%)	NA	NA	NA
**Mortality**	38/131 (29%)	44/131 (34%)	57/131 (44%)	68/129 (53%)
**Readmission** (survivors) [§]	NA	NA	9/72 (12%)	13/60 (22%)
**Hospital length of stay (LOS)**				
LOS (all participants), median days [IQR]	7 [4-11]	NA	NA	NA
LOS (survivors), median days [IQR]	8 [4-12]	NA	NA	NA
**mMRC**				
mMRC, median [IQR]	2 [1-4]	NA	NA	0 [0-1]
mMRC Grade 0	20/131 (15%)	NA	NA	41/61 (67%)
mMRC Grade 1	28/131 (21%)	NA	NA	13/61 (21%)
mMRC Grade 2	28/131 (21%)	NA	NA	6/61 (10%)
mMRC Grade 3	9/131 (7%)	NA	NA	0/61 (0%)
mMRC Grade 4	46/131 (35%)	NA	NA	1/61 (2%)
**Disability**				
With Disability	80/131 (61%)	NA	6/66 (9%)	NA
Without disability	51/131 (39%)	NA	60/66 (91%)	NA
**EQ5D-5L**				
HRQoL health utility score, median [IQR]	0.53 [0.26-0.66]	1 [0.8-1]	1 [0.81-1]	0.94 [0.8-1]
EQ5D-VAS, median [SD]	51.09 (16.53)	77.77 (19.29)	81.12 (18.94)	78.98 (17.48)
EQ5D Mobility				
No problems	8/131 (6%)	55/74 (74%)	51/66 (77%)	35/61 (57%)
Slight problems	33/131 (25%)	4/74 (5%)	6/66 (9%)	21/61 (34%)
Moderate problems	49/131 (37%)	8/74 (11%)	6/66 (9%)	4/61 (7%)
Severe problems	22/131 (17%)	5/74 (7%)	2/66 (3%)	1/61 (2%)
Unable to do	19/131 (15%)	2/74 (3%)	1/66 (2%)	0/61 (0%)
EQ5D Self care				
No problems	10/131 (8%)	55/74 (74%)	58/66 (88%)	45/61 (74%)
Slight problems	54/131 (41%)	9/74 (12%)	3/66 (5%)	12/61 (20%)
Moderate problems	37/131 (28%)	5/74 (7%)	2/66 (3%)	3/61 (5%)
Severe problems	17/131 (13%)	3/74 (4%)	1/66 (2%)	1/61 (2%)
Unable to do	13/131 (10%)	2/74 (3%)	2/66 (3%)	0/61 (0%)
EQ5D Usual activities				
No problems	4/131 (3%)	47/74 (64%)	47/66 (71%)	35/61 (57%)
Slight problems	22/131 (17%)	11/74 (15%)	10/66 (15%)	18/61 (30%)
Moderate problems	59/131 (45%)	10/74 (14%)	6/66 (9%)	7/61 (11%)
Severe problems	29/131 (22%)	5/74 (7%)	0/66 (0%)	1/61 (2%)
Unable to do	17/131 (13%)	1/74 (1%)	3/66 (5%)	0/61 (0%)
EQ5D Pain/Discomfort				
No symptoms	32/131 (24%)	57/74 (77%)	48/66 (73%)	46/61 (75%)
Slight symptoms	35/131 (27%)	10/74 (14%)	8/66 (12%)	9/61 (15%)
Moderate symptoms	48/131 (37%)	4/74 (5%)	9/66 (14%)	6/61 (10%)
Severe symptoms	13/131 (10%)	3/74 (4%)	1/66 (2%)	0/61 (0%)
Extreme symptoms	3/131 (2%)	0/74 (0%)	0/66 (0%)	0/61 (0%)
EQ5D Anxiety/Depression				
No symptoms	53/131 (40%)	58/74 (78%)	58/66 (88%)	49/61 (80%)
Slight symptoms	47/131 (36%)	11/74 (15%)	6/66 (9%)	9/61 (15%)
Moderate symptoms	21/131 (16%)	3/74 (4%)	1/66 (2%)	2/61 (3%)
Severe symptoms	10/131 (8%)	2/74 (3%)	1/66 (2%)	1/61 (2%)
Extreme symptoms	0/131 (0%)	0/74 (0%)	0/66 (0%)	0/61 (0%)

[§]Number of participants who survived to follow-up and had at least one hospital admission between the index admission and follow-up.

Table S 37. Demographic details, functional and clinical outcomes among participants with TB

TB	Hospital	Day 30	Day 90	1 Year
**Mean age, years [SD]**	51 [18]	NA	NA	NA
**Sex**				
Female	31/91 (34%)	NA	NA	NA
Male	60/91 (66%)	NA	NA	NA
**Mortality**	24/91 (26%)	28/91 (31%)	37/91 (41%)	41/87 (47%)
**Readmission** (survivors) [§]	NA	NA	3/52 (6%)	8/46 (17%)
**Hospital length of stay (LOS)**				
LOS (all participants), median days [IQR]	7 [3.5-10]	NA	NA	NA
LOS (survivors), median days [IQR]	8 [5-11.5]	NA	NA	NA
**mMRC**				
mMRC, median [IQR]	2 [1-4]	NA	NA	0 [0-1]
mMRC Grade 0	16/91 (18%)	NA	NA	30/46 (65%)
mMRC Grade 1	24/91 (26%)	NA	NA	10/46 (22%)
mMRC Grade 2	17/91 (19%)	NA	NA	6/46 (13%)
mMRC Grade 3	5/91 (5%)	NA	NA	0/46 (0%)
mMRC Grade 4	29/91 (32%)	NA	NA	0/46 (0%)
**Disability (Washington Group Set on Functioning)**				
With Disability	53/91 (58%)	NA	3/48 (6%)	NA
Without disability	38/91 (42%)	NA	45/48 (94%)	NA
**EQ5D-5L**				
HRQoL health utility score, median [IQR]	0.47 [0.13-0.65]	1 [0.74-1]	0.92 [0.78-1]	0.92 [0.74-1]
EQ5D-VAS, median [SD]	50.68 (16.4)	75.91 (19.53)	80.71 (16.06)	79.07 (16.94)
EQ5D Mobility				
No problems	5/91 (5%)	37/55 (67%)	31/48 (65%)	28/46 (61%)
Slight problems	22/91 (24%)	7/55 (13%)	10/48 (21%)	12/46 (26%)
Moderate problems	31/91 (34%)	8/55 (15%)	5/48 (10%)	4/46 (9%)
Severe problems	14/91 (15%)	2/55 (4%)	2/48 (4%)	2/46 (4%)
Unable to do	19/91 (21%)	1/55 (2%)	0/48 (0%)	0/46 (0%)
EQ5D Self care				
No problems	4/91 (4%)	40/55 (73%)	39/48 (81%)	32/46 (70%)
Slight problems	38/91 (42%)	6/55 (11%)	5/48 (10%)	9/46 (20%)
Moderate problems	25/91 (27%)	7/55 (13%)	2/48 (4%)	4/46 (9%)
Severe problems	10/91 (11%)	2/55 (4%)	2/48 (4%)	1/46 (2%)
Unable to do	14/91 (15%)	0/55 (0%)	0/48 (0%)	0/46 (0%)
EQ5D Usual activities				
No problems	4/91 (4%)	33/55 (60%)	32/48 (67%)	23/46 (50%)
Slight problems	14/91 (15%)	9/55 (16%)	9/48 (19%)	17/46 (37%)
Moderate problems	35/91 (38%)	8/55 (15%)	5/48 (10%)	5/46 (11%)
Severe problems	18/91 (20%)	5/55 (9%)	1/48 (2%)	1/46 (2%)
Unable to do	20/91 (22%)	0/55 (0%)	1/48 (2%)	0/46 (0%)
EQ5D Pain/Discomfort				
No symptoms	21/91 (23%)	40/55 (73%)	32/48 (67%)	31/46 (67%)
Slight symptoms	25/91 (27%)	10/55 (18%)	8/48 (17%)	9/46 (20%)
Moderate symptoms	33/91 (36%)	5/55 (9%)	8/48 (17%)	6/46 (13%)
Severe symptoms	10/91 (11%)	0/55 (0%)	0/48 (0%)	0/46 (0%)
Extreme symptoms	2/91 (2%)	0/55 (0%)	0/48 (0%)	0/46 (0%)
EQ5D Anxiety/Depression				
No symptoms	33/91 (36%)	48/55 (87%)	41/48 (85%)	35/46 (76%)
Slight symptoms	37/91 (41%)	5/55 (9%)	5/48 (10%)	7/46 (15%)
Moderate symptoms	14/91 (15%)	2/55 (4%)	1/48 (2%)	3/46 (7%)
Severe symptoms	7/91 (8%)	0/55 (0%)	1/48 (2%)	1/46 (2%)
Extreme symptoms	0/91 (0%)	0/55 (0%)	0/48 (0%)	0/46 (0%)

[§]Number of participants who survived to follow-up and had at least one hospital admission between the index admission and follow-up.

Section 8. Diagnostic accuracy supplemental data

Figure S 9. Diagnostic accuracy of biomarkers for heart failure and pneumonia: receiver operator curves

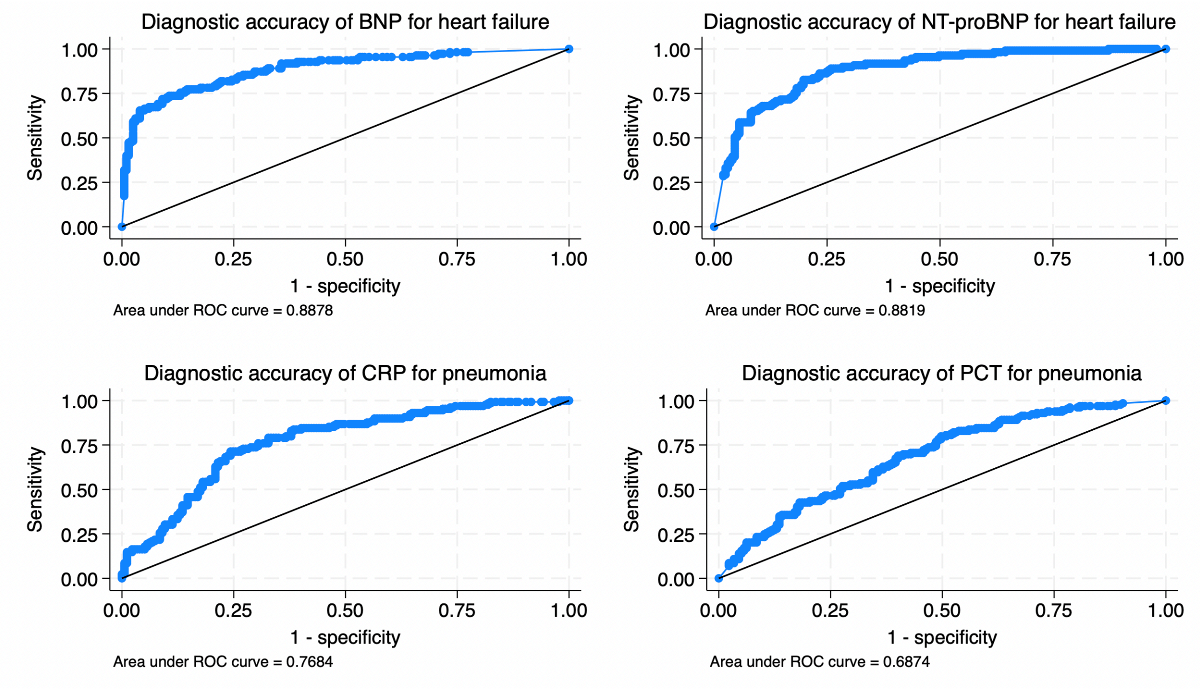

Table S 38. Diagnostic accuracy of natriuretic peptides for heart failure diagnosis

Biomarker cut point	TP	TN	FP	FN	Sensitivity (95% CI)	Specificity (95% CI)	PPV (95% CI)	NPV (95% CI)	PLR (95% CI)	NLR (95% CI)	OR (95% CI)	AUC (95% CI)
**BNP, n=309**					-	-	-	-	-	-	-	**0.89 (0.85-0.93)**
BNP 100	**102**	**121**	***78***	***8***	***92.7 (86.2 - 96.8)***	***60.8 (53.7 - 67.6)***	***56.7 (49.1 - 64.0)***	***93.8 (88.1 - 97.3)***	***2.4 (2.0 - 2.8)***	***0.1 (0.1 - 0.2)***	***19.8 (9.2 - 42.2)***	-
BNP 400	85	168	31	25	77.3 (68.3 - 84.7)	84.4 (78.6 - 89.2)	73.3 (64.3 - 81.1)	87.0 (81.5 - 91.4)	5.0 (3.5 - 7.0)	0.3 (0.2 - 0.4)	18.4 (10.3 - 33.1)	-
BNP 450	82	173	26	28	74.5 (65.4 - 82.4)	86.9 (81.4 - 91.3)	75.9 (66.7 - 83.6)	86.1 (80.5 - 90.5)	5.7 (3.9 - 8.3)	0.3 (0.2 - 0.4)	19.5 (10.8 - 35.2)	-
BNP 500	81	176	23	29	73.6 (64.4 - 81.6)	88.4 (83.2 - 92.5)	77.9 (68.7 - 85.4)	85.9 (80.3 - 90.3)	6.4 (4.3 - 9.5)	0.3 (0.2 - 0.4)	21.4 (11.7 - 39.1)	-
**NT-proBNP, n=306**					-	-	-	-	-	-	-	**0.88 (0.84-0.92)**
NT-proBNP 300	***107***	***75***	***122***	***2***	***98.2 (93.5 - 99.8)***	***38.1 (31.3 - 45.2)***	***46.7 (40.1 - 53.4)***	***97.4 (90.9 - 99.7)***	***1.6 (1.4 - 1.8)***	***0.0 (0.0 - 0.2)***	***32.9 (8.7 - .)***	-
NT-proBNP 400	106	84	113	3	97.2 (92.2 - 99.4)	42.6 (35.6 - 49.9)	48.4 (41.6 - 55.2)	96.6 (90.3 - 99.3)	1.7 (1.5 - 1.9)	0.1 (0.0 - 0.2)	26.3 (8.5 - 80.7)	-
NT-proBNP 900	100	117	80	9	91.7 (84.9 - 96.2)	59.4 (52.2 - 66.3)	55.6 (48.0 - 62.9)	92.9 (86.9 - 96.7)	2.3 (1.9 - 2.7)	0.1 (0.1 - 0.3)	16.2 (7.9 - 33.6)	-
NT-proBNP 1800	98	139	58	11	89.9 (82.7 - 94.9)	70.6 (63.7 - 76.8)	62.8 (54.7 - 70.4)	92.7 (87.3 - 96.3)	3.1 (2.4 - 3.8)	0.1 (0.1 - 0.3)	21.4 (10.7 - 42.3)	-

Cut points were identified from existing literature.[24] *Cut-points in bold italic reflect recommended cut-points from internationally-accepted guidelines* [4] The higher BNP threshold of 500 pg/mL, identified to enhance specificity, is also supported in the literature.[24]

TP: true positive; TN: true negative; FP: false positive; FN: false negative. PPV: positive predictive value; NPV: negative predictive value; PLR: positive likelihood ratio; NLR: negative likelihood ratio; OR: diagnostics odds ratio; AUC: area under receiver operator characteristics curve.

Table S 39. Diagnostic accuracy of C-reactive peptide and procalcitonin for pneumonia diagnosis

Biomarker cut point	TP	TN	FP	FN	Sensitivity (95% CI)	Specificity (95% CI)	PPV (95% CI)	NPV (95% CI)	PLR (95% CI)	NLR (95% CI)	OR (95% CI)	AUC (95% CI)
**CRP, n=306**					-	-	-	-	-	-	-	**0.77 (0.72-0.82)**
CRP 10	122	56	121	7	94.6 (89.1 - 97.8)	31.6 (24.9 - 39.0)	50.2 (43.7 - 56.7)	88.9 (78.4 - 95.4)	1.4 (1.2 - 1.5)	0.2 (0.1 - 0.4)	8.1 (3.6 - 18.0)	-
CRP 25	***112***	***83***	***94***	***17***	***86.8 (79.7 - 92.1)***	***46.9 (39.4 - 54.5)***	***54.4 (47.3 - 61.3)***	***83.0 (74.2 - 89.8)***	***1.6 (1.4 - 1.9)***	***0.3 (0.2 - 0.4)***	***5.8 (3.2 - 10.4)***	**-**
CRP 40	109	102	75	20	84.5 (77.1 - 90.3)	57.6 (50.0 - 65.0)	59.2 (51.8 - 66.4)	83.6 (75.8 - 89.7)	2.0 (1.7 - 2.4)	0.3 (0.2 - 0.4)	7.4 (4.2 - 13.0)	-
CRP 50	104	110	67	25	80.6 (72.7 - 87.0)	62.1 (54.6 - 69.3)	60.8 (53.1 - 68.2)	81.5 (73.9 - 87.6)	2.1 (1.7 - 2.6)	0.3 (0.2 - 0.5)	6.8 (4.0 - 11.6)	-
**PCT, n=306**					-	-	-	-	-	-	-	**0.69 (0.63-0.75)**
PCT 0.1	114	66	111	15	88.4 (81.5 - 93.3)	37.3 (30.1 - 44.9)	50.7 (43.9 - 57.4)	81.5 (71.3 - 89.2)	1.4 (1.2 - 1.6)	0.3 (0.2 - 0.5)	4.5 (2.4 - 8.3)	-
PCT 0.25	***93***	***96***	***81***	***36***	***72.1 (63.5 - 79.6)***	***54.2 (46.6 - 61.7)***	***53.4 (45.7 - 61.0)***	***72.7 (64.3 - 80.1)***	***1.6 (1.3 - 1.9)***	***0.5 (0.4 - 0.7)***	***3.1 (1.9 - 5.0)***	**-**
PCT 0.50	82	110	67	47	63.6 (54.6 - 71.9)	62.1 (54.6 - 69.3)	55.0 (46.7 - 63.2)	70.1 (62.2 - 77.1)	1.7 (1.3 - 2.1)	0.6 (0.5 - 0.8)	2.9 (1.8 - 4.6)	-
PCT 1.0	68	125	52	61	52.7 (43.7 - 61.6)	70.6 (63.3 - 77.2)	56.7 (47.3 - 65.7)	67.2 (60.0 - 73.9)	1.8 (1.4 - 2.4)	0.7 (0.5 - 0.8)	2.7 (1.7 - 4.3)	-

Cut points were identified from existing literature; *cut-points in bold italic reflect recommended thresholds from existing literature.*[25-27]

TP: true positive; TN: true negative; FP: false positive; FN: false negative. PPV: positive predictive value; NPV: negative predictive value; PLR: positive likelihood ratio; NLR: negative likelihood ratio; OR: diagnostics odds ratio; AUC: area under receiver operator characteristics curve.

Figure S 10. Decision curve analysis of BNP (cut point 100 pg/mL) for heart failure

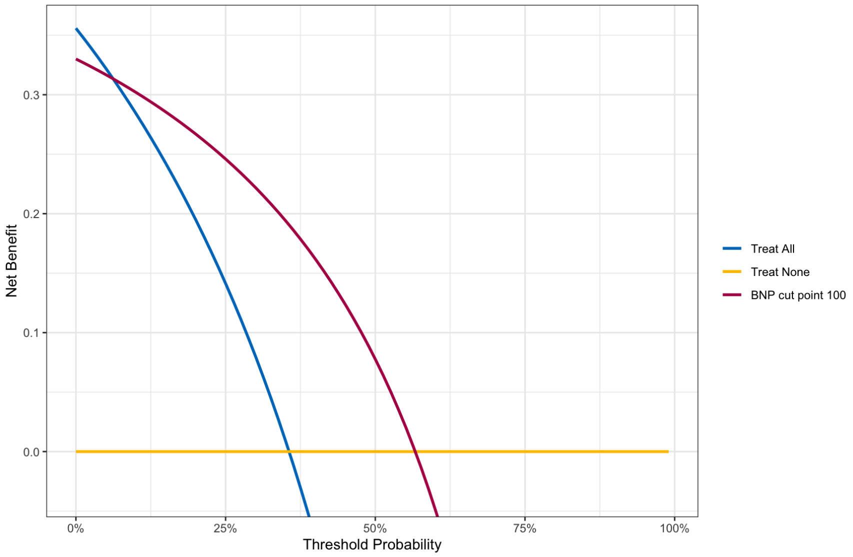

Figure S 11. Decision curve analysis of CRP (cut point 25 mg/l) for pneumonia

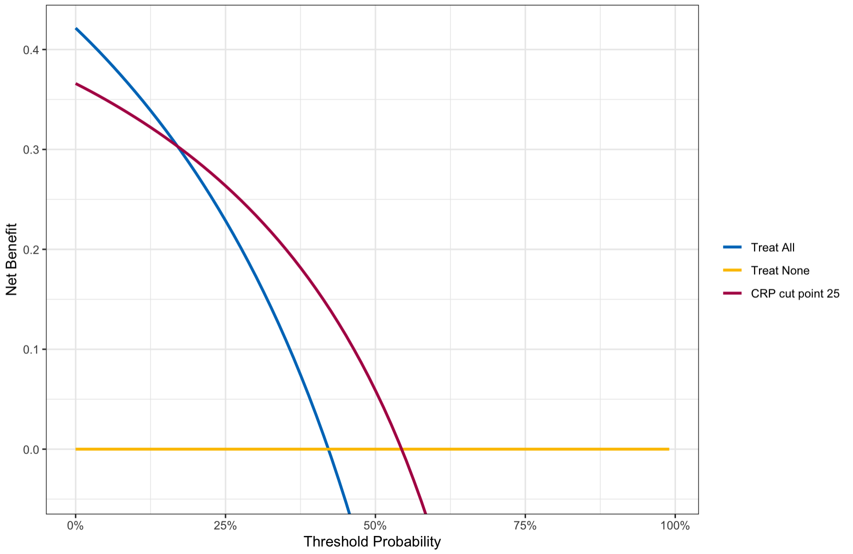

Figure S 12. Decision curve analysis of PCT (cut point 0.25ng/ml) for pneumonia

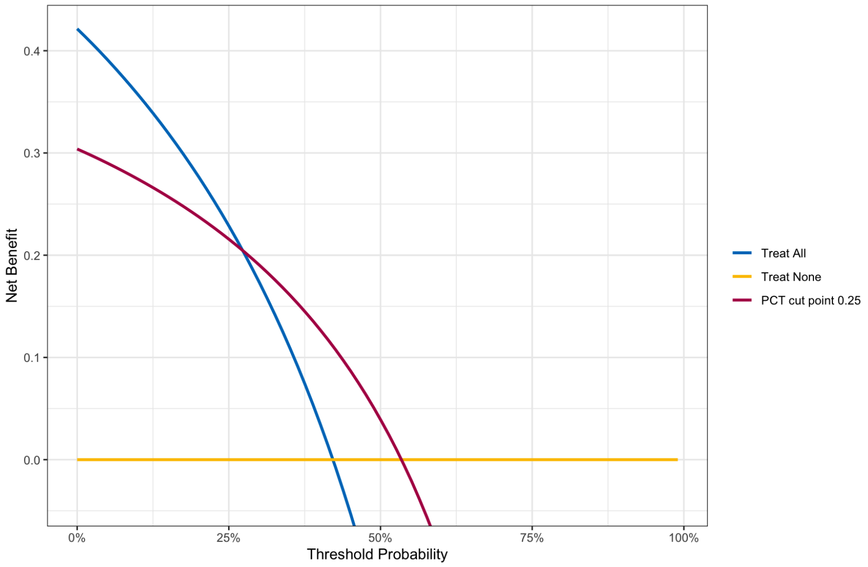

References

1. Mandell LA, Wunderink RG, Anzueto A, et al. Infectious Diseases Society of America/American Thoracic Society consensus guidelines on the management of community-acquired pneumonia in adults. *Clin Infect Dis* 2007; **44 Suppl 2**(Suppl 2): S27-72.
2. Lewis JM, Mphasa M, Keyala L, et al. A Longitudinal, Observational Study of Etiology and Long-Term Outcomes of Sepsis in Malawi Revealing the Key Role of Disseminated Tuberculosis. *Clin Infect Dis* 2022; **74**(10): 1840-9.
3. Mukoka M, Twabi HH, Msefula C, et al. Utility of Xpert MTB/RIF Ultra and digital chest radiography for the diagnosis and treatment of TB in people living with HIV: a randomised controlled trial (XACT-TB). *Trans R Soc Trop Med Hyg* 2023; **117**(1): 28-37.
4. Bozkurt B, Coats AJS, Tsutsui H, et al. Universal definition and classification of heart failure: a report of the Heart Failure Society of America, Heart Failure Association of the European Society of Cardiology, Japanese Heart Failure Society and Writing Committee of the Universal Definition of Heart Failure: Endorsed by the Canadian Heart Failure Society, Heart Failure Association of India, Cardiac Society of Australia and New Zealand, and Chinese Heart Failure Association. *Eur J Heart Fail* 2021; **23**(3): 352-80.
5. Harkness A, Ring L, Augustine DX, Oxborough D, Robinson S, Sharma V. Normal reference intervals for cardiac dimensions and function for use in echocardiographic practice: a guideline from the British Society of Echocardiography. *Echo Res Pract* 2020; **7**(1): X1.
6. Kou S, Caballero L, Dulgheru R, et al. Echocardiographic reference ranges for normal cardiac chamber size: results from the NORRE study. *Eur Heart J Cardiovasc Imaging* 2014; **15**(6): 680-90.
7. Robinson S, Rana B, Oxborough D, et al. A practical guideline for performing a comprehensive transthoracic echocardiogram in adults: the British Society of Echocardiography minimum dataset. *Echo Res Pract* 2020; **7**(4): G59-G93.
8. Robinson S, Ring L, Oxborough D, et al. The assessment of left ventricular diastolic function: guidance and recommendations from the British Society of Echocardiography. *Echo Res Pract* 2024; **11**(1): 16.
9. Spencer SA, Malowa F, McCarty D, et al. Causes, outcomes and diagnosis of acute breathlessness hospital admissions in Malawi: protocol for a multicentre prospective cohort study. *Wellcome Open Res* 2024; **9**: 205.
10. Thygesen K, Alpert JS, Jaffe AS, et al. Fourth Universal Definition of Myocardial Infarction (2018). *Circulation* 2018; **138**(20): e618-e51.
11. Mendis S, Thygesen K, Kuulasmaa K, et al. World Health Organization definition of myocardial infarction: 2008-09 revision. *Int J Epidemiol* 2011; **40**(1): 139-46.
12. Stanojevic S, Kaminsky DA, Miller MR, et al. ERS/ATS technical standard on interpretive strategies for routine lung function tests. *Eur Respir J* 2022; **60**(1).
13. Bakke PS, Ronmark E, Eagan T, et al. Recommendations for epidemiological studies on COPD. *Eur Respir J* 2011; **38**(6): 1261-77.
14. Reddel HK, Taylor DR, Bateman ED, et al. An official American Thoracic Society/European Respiratory Society statement: asthma control and exacerbations: standardizing endpoints for clinical asthma trials and clinical practice. *Am J Respir Crit Care Med* 2009; **180**(1): 59-99.
15. Haemoglobin concentrations for the diagnosis of anaemia and assessment of severity. Vitamin and Mineral Nutrition Information System. WHO/NMH/NHD/MNM/11.1. Geneva, Switzerland: World Health Organization, 2011.
16. Konstantinides SV, Meyer G, Becattini C, et al. 2019 ESC Guidelines for the diagnosis and management of acute pulmonary embolism developed in collaboration with the European Respiratory Society (ERS). *Eur Heart J* 2020; **41**(4): 543-603.
17. British Thoracic Society Standards of Care Committee Pulmonary Embolism Guideline Development G. British Thoracic Society guidelines for the management of suspected acute pulmonary embolism. *Thorax* 2003; **58**(6): 470-83.
18. Falster C, Hellfritzsch M, Gaist TA, et al. Comparison of international guideline recommendations for the diagnosis of pulmonary embolism. *Lancet Haematol* 2023; **10**(11): e922-e35.
19. Augustine DX, Coates-Bradshaw LD, Willis J, et al. Echocardiographic assessment of pulmonary hypertension: a guideline protocol from the British Society of Echocardiography. *Echo Res Pract* 2018; **5**(3): G11-G24.
20. MacDuff A, Arnold A, Harvey J, Group BTSPDG. Management of spontaneous pneumothorax: British Thoracic Society Pleural Disease Guideline 2010. *Thorax* 2010; **65 Suppl 2**: ii18-31.
21. Volpicelli G, Elbarbary M, Blaivas M, et al. International evidence-based recommendations for point-of-care lung ultrasound. *Intensive Care Med* 2012; **38**(4): 577-91.
22. Pisani L, De Nicolo A, Schiavone M, et al. Lung Ultrasound for Detection of Pulmonary Complications in Critically Ill Obstetric Patients in a Resource-Limited Setting. *Am J Trop Med Hyg* 2020; **104**(2): 478-86.
23. Fysh ETH, Smallbone P, Mattock N, et al. Clinically Significant Pleural Effusion in Intensive Care: A Prospective Multicenter Cohort Study. *Crit Care Explor* 2020; **2**(1): e0070.
24. Mueller C, Scholer A, Laule-Kilian K, et al. Use of B-type natriuretic peptide in the evaluation and management of acute dyspnea. *N Engl J Med* 2004; **350**(7): 647-54.
25. Dark P, Hossain A, McAuley DF, et al. Biomarker-Guided Antibiotic Duration for Hospitalized Patients With Suspected Sepsis: The ADAPT-Sepsis Randomized Clinical Trial. *JAMA* 2024.
26. Muller B, Harbarth S, Stolz D, et al. Diagnostic and prognostic accuracy of clinical and laboratory parameters in community-acquired pneumonia. *BMC Infect Dis* 2007; **7**: 10.
27. Garcia Vazquez E, Martinez JA, Mensa J, et al. C-reactive protein levels in community-acquired pneumonia. *Eur Respir J* 2003; **21**(4): 702-5.
