# Supplementary material for: Acute breathlessness as a cause of hospitalisation in Malawi: a prospective, patient-centred study to evaluate causes and outcomes
Source: Thorax. 2025 Sep 10;81(4):e223623. doi: 10.1136/thorax-2025-223623 (PMC13018744; doi:10.1136/thorax-2025-223623)
Supplement: online supplemental file 2 [file thorax-81-4-s002.docx]

**Reflexivity Statement:** Burden, causes and one-year health outcomes after hospitalisation with breathlessness in Malawi: a prospective multicentre cohort with a nested diagnostic accuracy study

| **Study conceptualization** | 1. **How does this study address local research and policy priorities?**   Admission to hospital due to breathlessness carries a significant burden to patients and healthcare systems, those living in low-income countries. Starting treatment is crucial to improve patient outcomes, but this relies on accurate diagnostic tests which are of limited availability in resource-constrained settings. We will provide an accurate description of acute breathlessness presentations in a multicentre prospective cohort study in Malawi, a low resource setting in sub-Saharan Africa, and examine approaches that may improve diagnostic accuracy. |
| --- | --- |
|  | 1. **How were local researchers involved in study design?**   Local researchers are co-authors and have contributed to study design from project conception. |
| **Research management** | 1. **How has funding been used to support the local research team(s)?**   Research funding has been used to employ research teams and build local clinical research capacity. |
| **Data acquisition and analysis** | 1. **How are research staff who conducted data collection acknowledged?**   We have described author contributions using CRediT criteria, explicitly acknowledging contributions in the manuscript. All contributing researchers have been listed as authors or collaborators on the manuscript. |
|  | 1. **How have members of the research partnership been provided with access to study data?**   Members of the research partnership were provided with access to study data through the Malawi Liverpool Wellcome (MLW) Research Programme data portal |
|  | 1. **How were data used to develop analytical skills within the partnership?**   Data were discussed in detail at fortnightly Lung Health research meetings at MLW, through regular Multilink PhD meetings, MLW PhD meetings, and Multilink clinical workpackage meetings. These meetings provided a platform for discussion on data analytical approaches. In addition, data provided within this study will support a number of proposed Malawian MMed student projects. |
| **Data interpretation** | 1. **How have research partners collaborated in interpreting study data?**   See answer to 6. |
| **Drafting and revising for intellectual content** | 1. **How were research partners supported to develop writing skills?**   Within the collaboration, we invited members to join as authors of the manuscript within the ‘writing group’ or ‘collaborating group’. Those who wished to join the writing group were supported to provide input and critically review the manuscript. |
|  | 1. **How will research products be shared to address local needs?**   We have made presentations at appropriate research dissemination conferences, within our established networks, research instituions, to the stakeholders within the local hospitals (Queen Elizabeth Central Hospital and Chiradzulu District Hospital), and to a broader natiowide ‘grand round’ session. As part of this project, we also developed a diagnostic framework applicable to the hospital context in Malawi. This framework has been presented by several authors within our partnership to healthcare workers in Malawi, and with proposals to integrate this (together with the findings from this manuscript) within Medical School education in Malawi. |
| **Authorship** | 1. **How is the leadership, contribution and ownership of this work by LMIC researchers recognised within the authorship?**   Please refer to the section on “Authors’ contribution” in the manuscript. |
|  | 1. **How have early career researchers across the partnership been included within the authorship team?**   The first author is an early career researcher (enrolled as a clinical PhD student). Additionally, we have supported the clinical research team involved with data collection for the study join the writing group as authors within this manuscript. |
|  | 1. **How has gender balance been addressed within the authorship?**   8/23 of the authors are women; local LMIC (11/23) and HIC (12/23) settings. Their contribution to the study is acknowledged in the ‘Authors’ contribution’ section of the manuscript. We have also recognised all research collaborators who supported this study. |
| **Training** | 1. **How has the project contributed to training of LMIC researchers?**   See answer to question 3 and question 9. Formal research training provided at the start of the study on study Standard Operating Procedures; research training provided through prospective observation and feedback of research activities; fornightly meetings with the entire LMIC research team including opportunity to present at journal club and review/interpret data obtained through study activities. Training was provided on comprehensive echocardiography image acquisition and image interpretation; for for lung ultrasound acquisition and interpretation. Dat will contribute towards research training through support of MMed and MSc projects based in Malawi. |
| **Infrastructure** | 1. **How has the project contributed to improvements in local infrastructure?**   This project involves making enhanced commercially available CE marked diagnostic tests available in the collaborating hospital sites. All equipment was left free of charge to the local hospitals. Results from these tests were be made available to the clinical teams to inform care for patients. |
| **Governance** | 1. **What safeguarding procedures were used to protect local study participants and researchers?**   All researchers are qualified professionals. The researchers have attended safeguarding training and observed safeguarding policy procedures as per Malawi-Liverpool-Trust Clinical Research Programme and Liverpool School of Tropical Medicine when facilitating delivery of this project. |
