# Supplementary material for: Acute breathlessness as a cause of hospitalisation in Malawi: a prospective, patient-centred study to evaluate causes and outcomes
Source: Thorax. 2025 Sep 10;81(4):e223623. doi: 10.1136/thorax-2025-223623 (PMC13018744; doi:10.1136/thorax-2025-223623)
Supplement: online supplemental file 3 [file thorax-81-4-s003.pptx]

## Slide 1
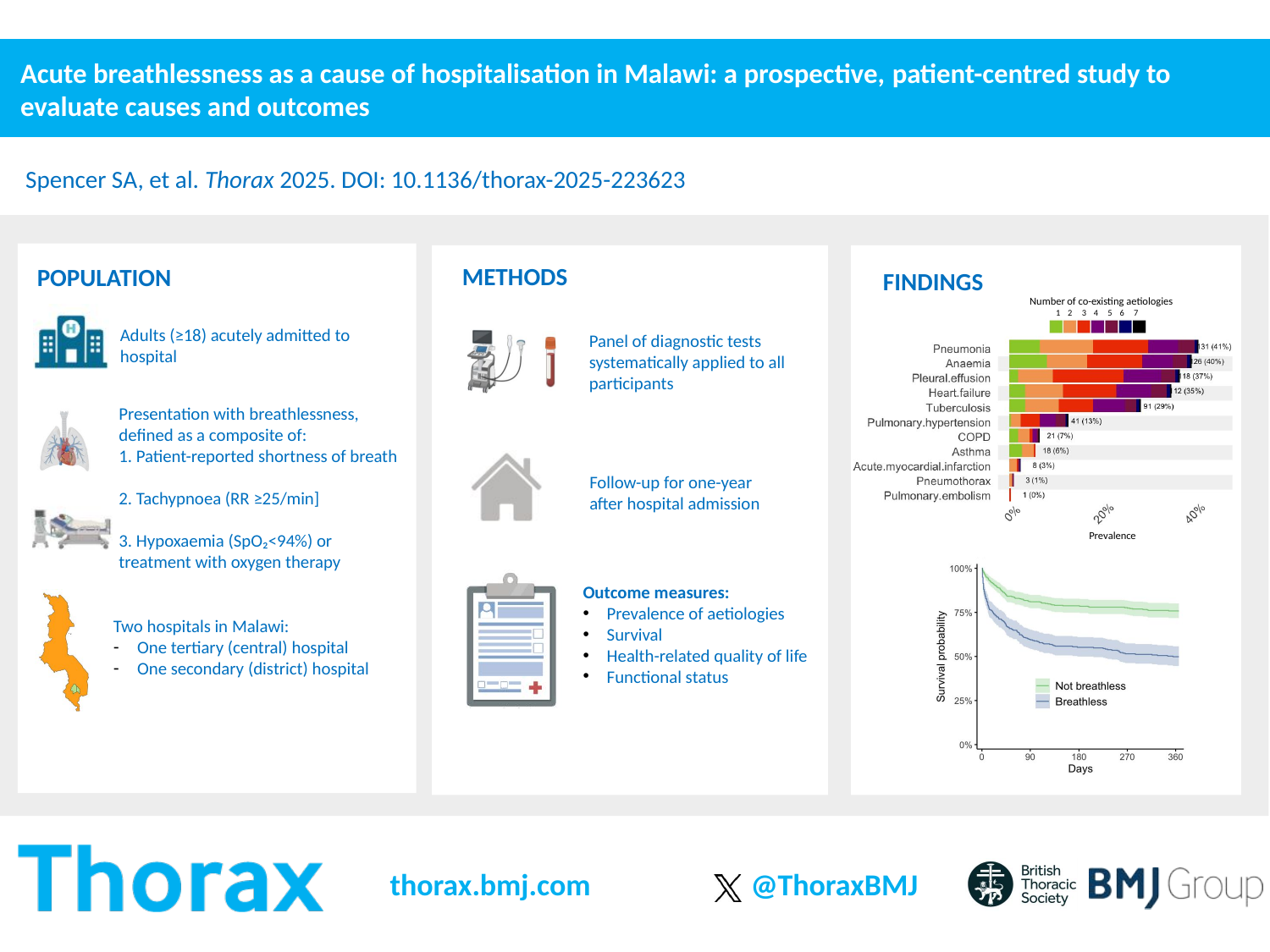

Acute breathlessness as a cause of hospitalisation in Malawi: a prospective, patient-centred study to evaluate causes and outcomes
Spencer SA, et al. Thorax 2025. DOI: 10.1136/thorax-2025-223623
METHODS
POPULATION
FINDINGS
Manuscript Title
Number of co-existing aetiologies
1 2 3 4 5 6 7
Adults (≥18) acutely admitted to hospital
Panel of diagnostic tests systematically applied to all participants
Presentation with breathlessness, defined as a composite of:
1. Patient-reported shortness of breath
2. Tachypnoea (RR ≥25/min]
3. Hypoxaemia (SpO₂<94%) or treatment with oxygen therapy
Follow-up for one-year after hospital admission
Prevalence
Outcome measures:
Prevalence of aetiologies
Survival
Health-related quality of life
Functional status
Two hospitals in Malawi:
One tertiary (central) hospital
One secondary (district) hospital
© Author(s) (or their employer(s) 2019. Re-use permitted under CC BY. Published by BMJ.
thorax.bmj.com @ThoraxBMJ
